# Downregulating PDPK1 and taking phillyrin as PDPK1-targeting drug protect hepatocytes from alcoholic steatohepatitis by promoting autophagy

**DOI:** 10.1038/s41419-022-05422-3

**Published:** 2022-11-23

**Authors:** Yuan Zhang, Yuhao Ding, Huizi Zhao, Zhonghao Wang, Fanle Zeng, Zhenzhen Qian, Jun Li, Taotao Ma, Cheng Huang

**Affiliations:** Inflammation and Immune Mediated Diseases Laboratory of Anhui Province, Anhui Institute of Innovative Drugs, School of Pharmacy, Anhui Medical University, Hefei, 230032 China

**Keywords:** Protein-protein interaction networks, Macroautophagy

## Abstract

The health risk stemming from drinking alcohol is serious, sometimes even life-threatening. Alcoholic steatohepatitis (ASH) is a critical stage leading to cirrhosis and end-stage liver disease. However, its pathogenesis is still far from clearly understood, and a treatment that is widely recognised as effective has not been discovered. Interestingly, PDPK1,3-phosphoinositide-dependent protein kinase 1, also known as PDK1, was observed to be obviously increased in the ASH model by our researchers. We also investigated the protective role of autophagy in ASH. Here, we studied the function of PDPK1 and found an efficient treatment to alleviate symptoms by targeting PDPK1 in ASH. In our study, PDPK1 affected hepatocyte self-healing by inhibiting autophagy. Both inhibiting PDPK1 and the phosphorylation of PDPK1 (ser241) could protect hepatocytes from suffering heavy alcoholic hepatitis.

## Introduction

A rising death toll of alcoholic liver disease (ALD) reveals such the phenomenon that liver injury has become a major threat to human health [1]. ALD refers to a series of liver diseases that result from liver damage caused by long-term and improper alcohol intake [2]. Initially manifested as marked hepatocellular steatosis, it can progress to alcoholic steatohepatitis (ASH), liver fibrosis, liver cirrhosis, and even hepatocellular carcinoma [3]. During the deteriorating course of ALD, ASH is often recognised as a critical stage leading to cirrhosis and end-stage liver disease. An effective treatment should be taken early during the ASH stage to prevent the aggravation of the liver disease. Although several treatments have been tried, such as alcohol withdrawal, corticosteroids, biologics (e.g., antitumour necrosis factor (TNF)-alpha and IL-1 receptor antagonist), and liver transplantation [4–6], a more practical, safer and widely accessed treatment option is needed.

Autophagy is an important cellular component degradation and energy-recycling process in the body [7, 8]. It is now acknowledged to participate in the pathogenesis of a number of diseases and is activated under a variety of stress conditions. Autophagy involves the formation of double-membrane autophagosomes that wrap around substrates and fuse with lysosomes for degradation [9]. The intracellular level of lipids can be regulated by autophagy through degrading lipid droplets [10]. Meanwhile, recent studies showed that ethanol intake could disorganise lipid metabolism and cause damage to organelles in ASH [11], suggesting that lipid droplets and damaged organelles may accumulate in the course of the disease. Recent articles have reported that autophagy can be activated when the body is exposed to alcohol [12, 13]. It is easy to draw such an assumption that modulating autophagy might provide a new strategy for the treatment of ASH.

PDPK1,3-phosphoinositide-dependent protein kinase 1, also known as PDK1, is a phosphorylation-regulated kinase that is expressed in various organs of eukaryotes and plays a central role in activating a variety of cell signalling pathways [14–16]. Studies have found that when cells are infected by external viruses, PDPK1 is released from the endoplasmic reticulum and translocated to the cell membrane, resulting in the phosphorylation of AKT and activation of the AKT-mTOR pathway, ultimately inhibiting autophagy [17]. Nevertheless, both the expression and the function of PDPK1 in ASH have not yet been reported. Further exploring the expression and function of PDPK1 may uncover the potential relationship between PDPK1 and ASH. This has aroused the extensive research enthusiasm of our researchers. Notably, we tested the expression of PDPK1 in ASH, and the level increased. The results indicated that PDPK1 may influence ASH progression. We further detected the autophagic flux under changes in PDPK1 to investigate whether PDPK1 could regulate cellular autophagy in ASH.

Then, we found a drug targeting PDPK1 by SPR to be a promising drug for ASH treatment. Surprisingly, phillyrin was demonstrated to target PDPK1 and inhibit the phosphorylation of PDPK1 (ser241). Phillyrin is a representative extract from Forsythia suspensa, which is a traditional Chinese herbaceous plant and certain functional foods. The effect of phillyrin has been tested on different kinds of diseases. Zhong et al. showed the anti-inflammatory action of phillyrin in lung injury [18]. Jiang et al. reported that phillyrin could inhibit the function of immune cells in the central nervous system (CNS) to prevent blood–brain barrier (BBB) damage [19]. Wang et al. claimed that phillyrin has an antifibrotic effect [20]. Moreover, it has been reported that phillyrin can attenuate high glucose-induced lipid accumulation [21]. Alcohol could also induce hepatic steatosis. However, it remains unclear whether phillyrin protects against ethanol-induced steatosis in vitro and in vivo. Thus, a series of experiments were performed to determine the protective function of phillyrin in ASH. As expected, the therapeutic effect of phillyrin was confirmed according to positive results. In summary, our study offered a new strategy for ASH treatment: Inhibiting PDPK1 and the phosphorylation of PDPK1 (ser241) to protect hepatocytes from alcoholic steatosis by promoting autophagy.

## Results

### Autophagy mitigated ethanol-induced liver injury and steatosis in ASH

**Fig. 1:**
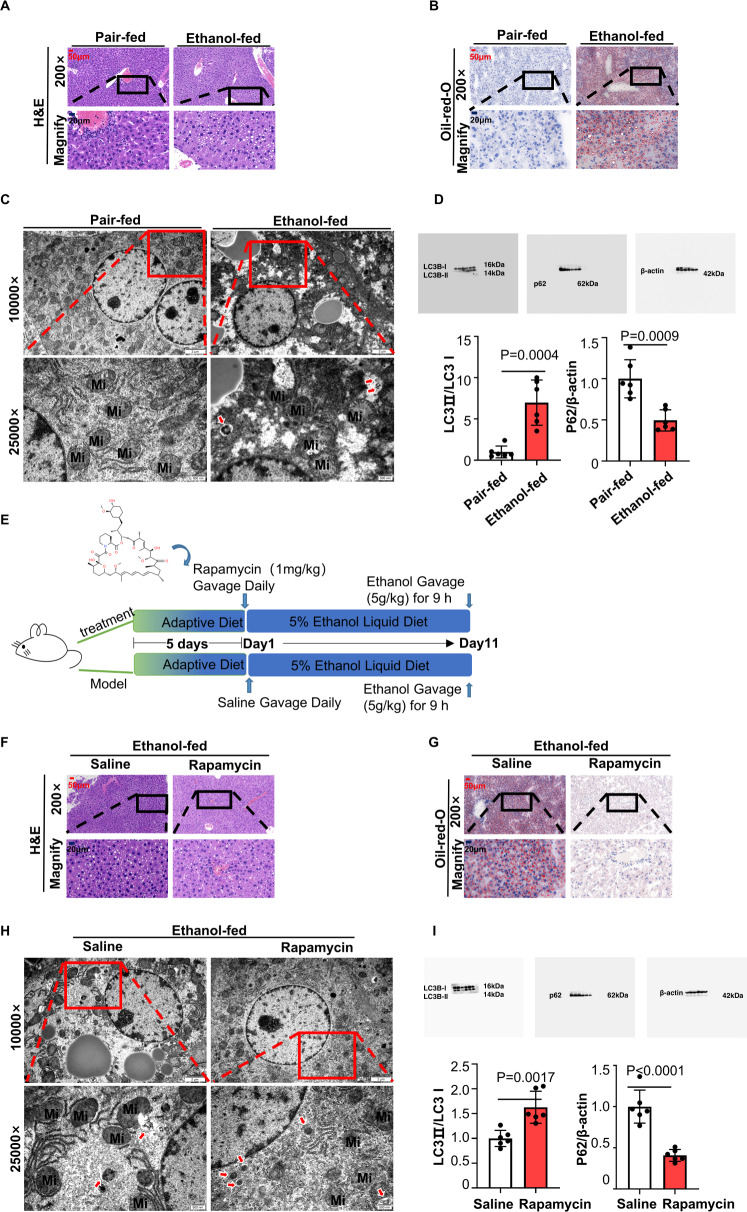
Autophagy mitigated ethanol-induced liver injury and steatosis in ASH. **A** H&E staining of liver tissues from two groups, including CD-fed mice and ethanol-fed mice (*n* = 6). Scale bar, 50 μM, magnification, 200×; Magnified pictures, scale bar, 20 μM. **B** Oil red O staining of liver tissues from the two groups, The pictures are sorted the same as the H&E staining in Fig. 1A (*n* = 6). Scale bar, 50 μM, magnification, 200×; Magnified pictures, scale bar, 20 μM. **C** Representative images of liver tissue captured by transmission electron microscopy (*n* = 6). Autophagosomes are marked by red arrows. Mi is the abbreviation for mitochondria. LD is the abbreviation of lipid droplet. Scale bar, 2 μM, magnification, 1000×; scale bar, 500 nM, magnification, 2500×. **D** Immunoblot of liver tissue lysates from the CD-fed group and ethanol-treated group (*n* = 6). Quantification of the ratios of LC3II/LC3I and p62/β-actin. **E** The time, dosage and form of rapamycin administration by adopting the Gao-Binge protocol to establish an animal model in C57BL/6 mice. (6–8 mice per group). **F** H&E staining of liver tissues from two groups, including saline-treated mice and rapamycin-treated mice (*n* = 6). Scale bar, 50 μM, magnification, 200×; Magnified pictures, scale bar, 20 μM. **G** Oil red O staining of liver tissues from the two groups. The pictures are sorted the same as the H&E staining in Fig. 1F (*n* = 6). Scale bar, 50 μM, magnification, 200×; Magnified pictures, scale bar, 20 μM. **H** Representative image of liver tissue captured by transmission electron microscopy (*n* = 6). Autophagosomes are marked by red arrows. Mi is the abbreviation of mitochondria. LD is the abbreviation for lipid droplets. Scale bar, 2 μM, magnification, 1000×; scale bar, 500 nM, magnification, 2500×. **I** Immunoblot of liver tissue lysates from the saline-treated group and rapamycin-treated group (*n* = 6). Quantification of the ratios of LC3II/LC3I and p62/β-actin. Data represent the mean ± SEM of six biological replicates per condition. Each dot represents a mouse. *P* < 0.05 was considered statistically significant. NS indicates no significance (two-tailed *t*-tests).

According to many convincing articles [22, 23], the metabolism of lipids can be disorganised by ethanol in ASH patients’ livers. Lipid droplets were always largely accumulated in those patients’ hepatocytes and could not be degraded efficiently. Many studies have tried to regulate the degradation of lipids to alleviate ASH. Known as a cellular self-eating process, autophagy was reported to be involved in the digestion of lipid droplets [10, 24]. However, autophagy is usually recognised as a double-edged sword in current reports [25]. To investigate whether autophagy could occur in ASH and which kind of role autophagy plays in ASH (positive or negative), we established an ASH model by adopting the NIAAA model protocol [26]. As shown in Fig. 1A, liver biopsies stained with haematoxylin-eosin from ethanol-fed mice are full of lipid vacuoles. In Fig. 1B, lipid droplets stained with oil Red O showed the model group mice suffering from dyslipidaemia. The levels of serum AST and ALT increased in the model group, indicating an abnormal liver function in model mice (Supplementary Fig. 1A, B). The levels of serum TG and T-CHO increased in the model group, indicating an altered lipid profile in the blood (Supplementary Fig. 1C, D). Transmission electron microscopy (TEM) of ultrathin liver sections showed that the number of autophagosomes in the model group was greater than that in the normal group (Fig. 1C), suggesting that autophagy could be activated in ethanol-treated mice. Another autophagy index, the protein level of soluble microtubule-associated protein light chain 3 (LC3)-II/LC3I (upregulated) and p62/β-actin (downregulated), drew the same conclusion: Autophagy-flux was induced in the model group (Fig. 1D). Next, we further tested the function of autophagy by using the autophagy-inducer rapamycin. We listed the time, dosage and form of medication administration in Fig. 1E. The decrease in lipid vacuoles in H&E staining and red lipid droplets in oil Red O staining (Fig. 1F, G) showed that rapamycin helped ethanol-treated mice ease the symptoms from ethanol-induced dyslipidaemia. The levels of serum AST and ALT decreased in the rapamycin-treated group, indicating a recovery in liver function (Supplementary Fig. 1E, F). The levels of serum TG and T-CHO were lowered in the rapamycin-treated group, suggesting a recovery in blood lipid metabolism (Supplementary Fig. 1G, H). Consistent with former reports [27], our results also demonstrated that rapamycin could reduce hepatic steatosis in ethanol-treated mice. Meanwhile, the number of autophagosomes increased evidently in the rapamycin-treated group by TEM (Fig. 1H) and western blot (Fig. 1I). In this part, we tested whether autophagy could be activated by ethanol and promote cellular self-repair, that is, whether autophagy plays a protective role in ASH.

### PDPK1 affected hepatocyte self-healing by inhibiting autophagy

**Fig. 2:**
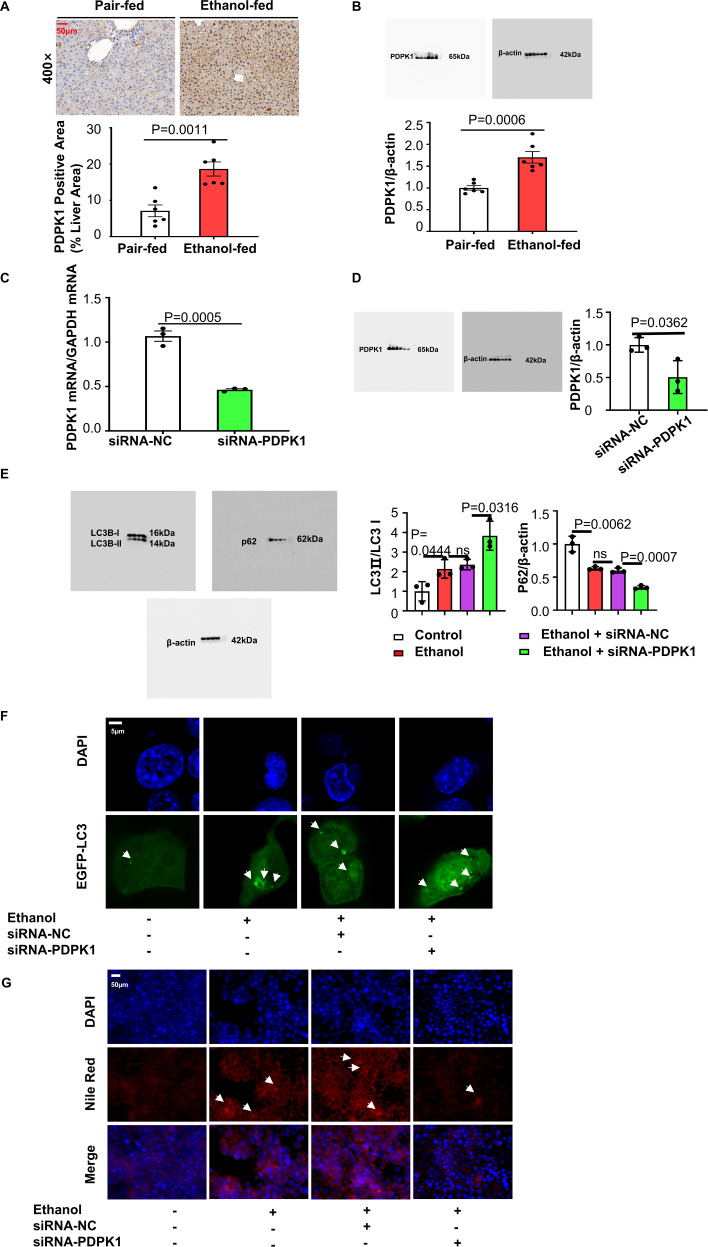
Downregulated PDPK1 expression attenuated steatosis and promotes autophagy in hepatocytes. **A** Positive immunostaining for PDPK1(a brown colour) was predominantly located in lipid-rich hepatocytes in ethanol-fed mice (*n* = 6). Scale bar, 50 μM, magnification, 400× Quantification of PDPK1 Positive Area (% Liver Area). **B** Immunoblot of liver tissue lysates from the CD-fed group and ethanol-treated group (*n* = 6). Quantification of the ratios of PDPK1/β-actin. **C** The efficiency of siRNA-PDPK1 detection by RT‒qPCR (*n* = 3). **D** Immunoblot of cell lysates. Quantification of the ratio of PDPK1/β-actin (*n* = 3). **E** Immunoblot of cell lysates. Quantification of the ratio of LC3II/LC3I and p62/β-actin (*n* = 3). **F** CLSM images of LC3 in AML-12 cells transfected with EGFP-LC3 plasmid (*n* = 3). Scale bar, 5 μM. **G** Nile red staining of lipids in AML-12 cells (*n* = 3). Scale bar, 50 μM, magnification, 400×. Data represent the mean ± SEM of three to six biological replicates per condition. Each dot represents a sample. *P* < 0.05 was considered statistically significant. NS indicates no significance (**A**–**D**, two-tailed *t*-tests; **E**, one-way ANOVA).

Since our former experiments showed that further promoting autophagy in ethanol-treated mice could alleviate ASH, we next detected whether autophagy was blocked in the process of activating self-protection. Interestingly, we found a higher expression of PDPK1 in ASH mouse livers than in normal livers from the results of immunohistochemical staining and western blotting (Fig. 2A, B). Additionally, scientists have reported that the increasing levels of PDPK1 could inhibit autophagy [28], suggesting a functional involvement of PDPK1 in autophagy in ASH mice. Then, we tried to regulate the level of PDPK1 in AML-12 cells to test this hypothesis. First, the level of PDPK1 was downregulated by transfection with siRNA. Figure. 2C, D show the efficiency of transfection, showing that siRNA successfully downregulated the PDPK1 mRNA and protein levels. Later, MTT assays were performed to choose the most suitable concentration for stimulation. Concentrations of ethanol over 100 mM in culture medium had a marked cytotoxic effect on AML-12 cells, and 200 mM ethanol was selected for use during the next experiments because of its stability (Supplementary Fig. 2). As expected, the autophagy-flux (the level of LC3II/LC3I increased, p62/β-actin decreased) was enhanced in the siRNA-transfected group compared with other groups (Fig. 2E). Confocal laser scanning microscopy (CLSM) also showed that GFP-LC3 points increased after 24 h of transfection with both siRNA and GFP-LC3 plasmid (Fig. 2F). To further investigate the function of PDPK1 in ASH, Nile red staining (Fig. 2G) was performed to detect the intracellular lipid vacuoles. Fewer lipid vacuoles were observed when PDPK1 was knocked down by siRNA, suggesting that AML-12 cells were protected after PDPK1 downregulation.

**Fig. 3:**
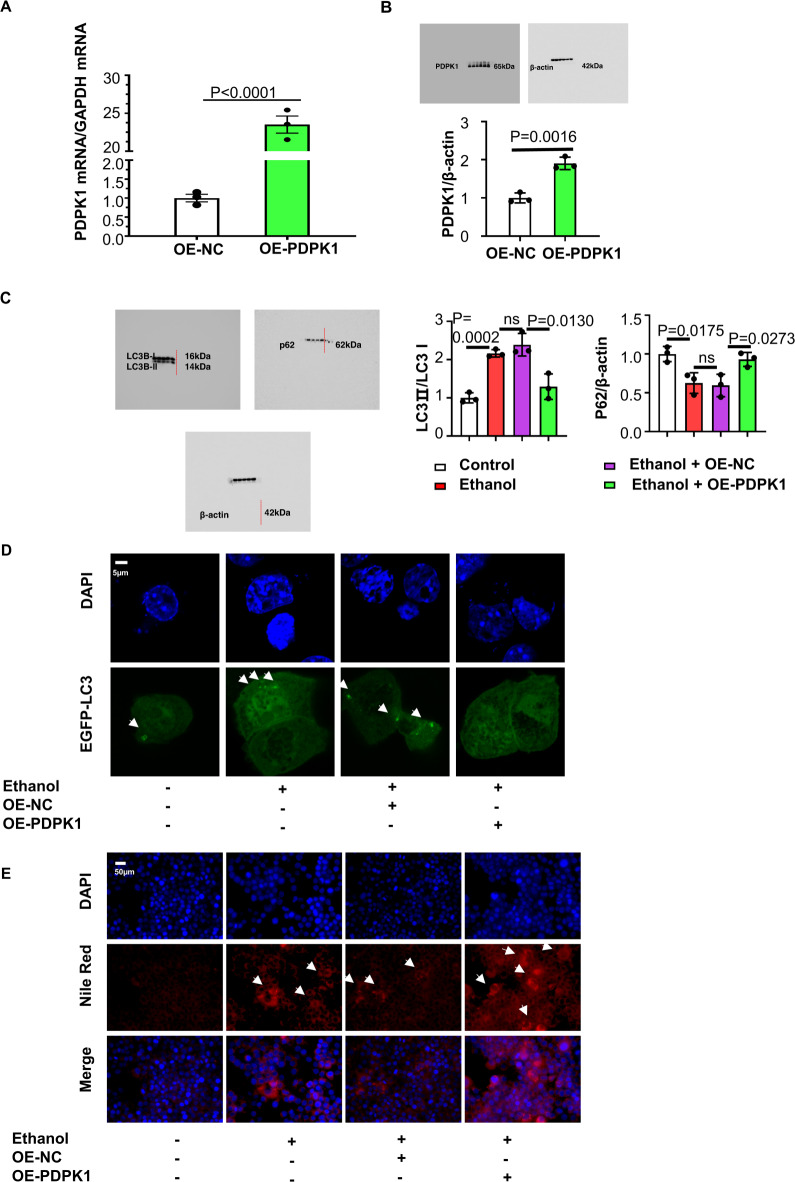
Upregulated PDPK1 expression promoted steatosis and inhibits autophagy in hepatocytes. **A** The efficiency of HBLV-m-PDPK1-3xflag-mCherry-PURO detected by RT-qPCR (*n* = 3). **B** Immunoblot of cell lysates. Quantification of the ratio of PDPK1/β-actin (*n* = 3). **C** Immunoblot of cell lysates. Quantification of the ratio of LC3II/LC3I and p62/β-actin (*n* = 3). **D** CLSM images of LC3 in AML-12 cells transfected with EGFP-LC3 plasmid (*n* = 3). Scale bar, 5 μM. **E** Nile red staining of lipids in AML-12 cells (*n* = 3). Scale bar, 50 μM, magnification, 400×. Data represent the mean ± SEM of three biological replicates per condition. Each dot represents a sample. *P* < 0.05 was considered statistically significant. NS indicates no significance (**A**, **B**, two-tailed *t*-tests; **C** one-way ANOVA).

Then, we constructed a PDPK1-overexpressing cell stable transfection line by transfection with HBLV-m-PDPK1-3xflag-mcherry-PURO. The transfection efficiency was detected by RT‒qPCR and immunoblotting (Fig. 3A, B). Both immunoblotting and CLSM showed that cellular autophagy-flux was inhibited by PDPK1 overexpression (Fig. 3C, D). Consistently, Nile red staining (Fig. 3E) confirmed that overexpression of PDPK1 could further increase the accumulated lipid vacuoles caused by ethanol.

### Pharmacologic inhibition of phosphorylation of PDPK1 (Ser241) by targeting PDPK1 could attenuate steatosis of hepatocytes by promoting its autophagy

**Fig. 4:**
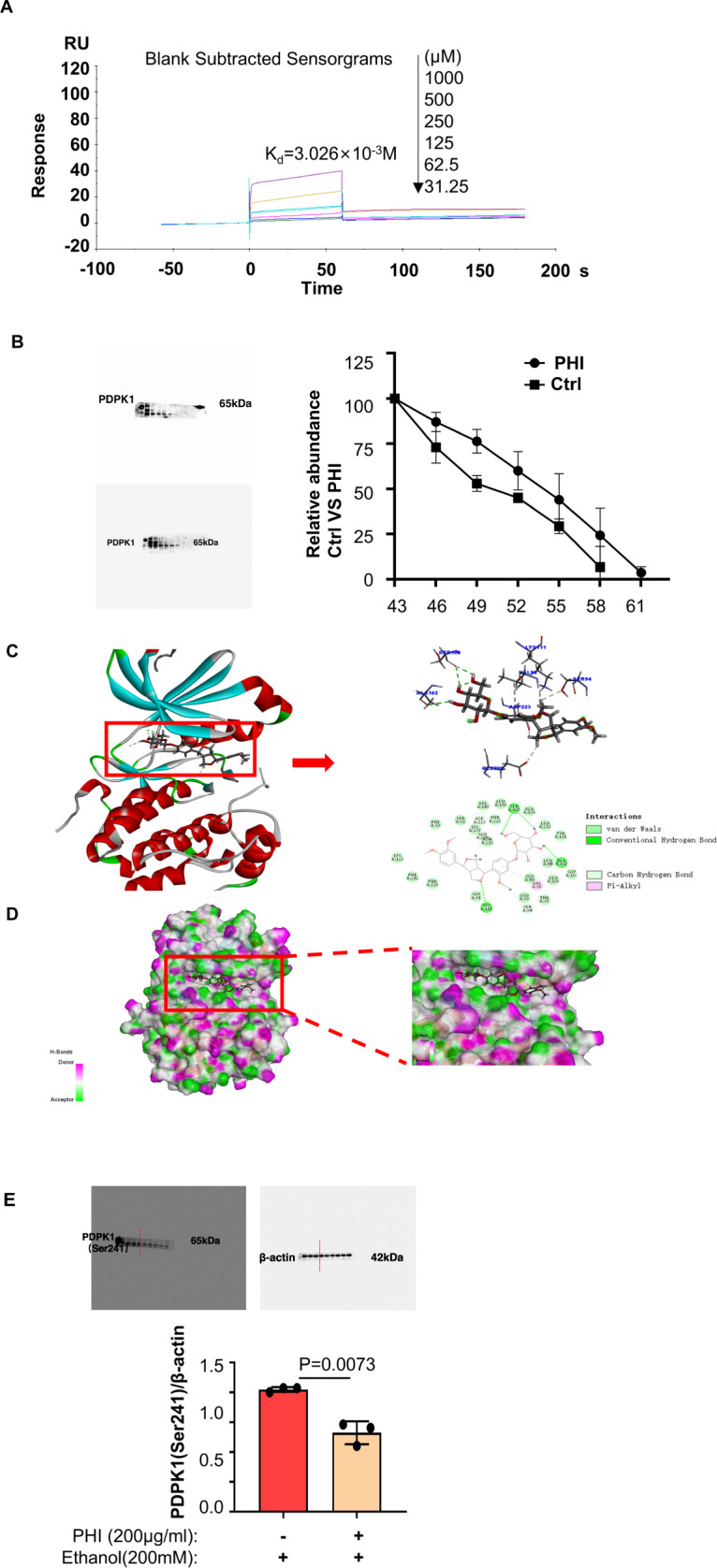
Phillyrin could target PDPK1 and inhibit the phosphorylation of PDPK1 (Ser241). Figures 4C, D were obtained by the PHASE module of Schrödinger’s molecular modelling software package. **A** The binding affinity of phillyrin to PDPK1 was detected by SPR. **B** AML-12 cells incubated with or without phillyrin (200 μg/ml) for 24 h were subjected to CETSA assay (*n* = 3). C Binding site of phillyrin to PDPK1. The chemical bonds that phillyrin binds to PDPK1 are marked in the figure. **D** 3D map of phillyrin occupying the PDPK1 pocket. **E** Immunoblot of cell lysates from the ethanol-induced group and ethanol-induced group treated with phillyrin (200 μg/ml) (3 representatives shown). Quantification of the ratio of PDPK1 (Ser241)/β-actin. Data represent the mean ± SEM of three biological replicates per condition. Each dot represents a sample. *P* < 0.05 was considered statistically significant. NS indicates no significance (**B** and **E**, two-tailed *t*-tests).

The importance of PDPK1 expression was demonstrated under the effect of ethanol. However, discovering an efficient drug target for PDPK1 may be something more urgent. Thus, research in our lab focused on which drug molecule could target with PDPK1. Luckily, phillyrin was confirmed to interact with PDPK1. A surface plasmon resonance (SPR) assay was carried out to measure the binding affinity of phillyrin with PDPK1 by using a Biacore T200. As shown in Fig. 4A (*K*_d_ = 3.026 × 10^−3^), phillyrin truly had a binding affinity to PDPK1 in a concentration-dependent manner. Additionally, a cellular thermal shift assay (CETSA) was performed to verify the interaction between phillyrin and PDPK1. According to the interaction principle, proteins tend to withstand high temperatures, triggering degradation when they bind with drugs [29]. As shown in Fig. 4B, PDPK1 almost disappeared at 55 °C in vehicle‐treated cells, while it disappeared at 58 °C in phillyrin-treated cells. Degradation curves obtained from three independent experiments intuitively showed the stability of phillyrin-treated samples. Furthermore, the PHASE module of Schrödinger’s molecular modelling software package was used to identify the underlying potential cellular targets of phillyrin. Accordingly, PDPK1 (3-phosphoinositide dependent protein kinase 1) was identified as a potential target of phillyrin after the intermolecular binding force analysis. Phillyrin can bind to amino acid residues (SER160, TYR161 and ALA162) in the active pocket of PDPK1. There are at least three hydrogen bonds that can be formed between the glycosyl of the phillyrin and the hinge region of the PDPK1 (Fig. 4C). The phenyl glycoside of phillyrin occupies the adenine region of the ATP activation pocket, and the furan ring occupies the ribose region; the terminal dimethoxyphenyl group is located in the triphosphate region, as shown in Fig. 4D. To some extent, the phosphorylated form of PDPK1 could be inhibited because the ATP-binding pocket of PDPK1 is being occupied by phillyrin. In addition, the immunoblotting analysis showed that the level of phosphorylation of PDPK1 at Ser241 was decreased only in phillyrin-treated (200 μg/ml) AML-12 cells (Fig. 4E). The concentration was selected by MTT from Supplementary Fig. 3A. Phillyrin scarcely affects cell viability over a wide range of concentrations according to the MTT assay, even at its maximum solubility. We also tested whether 50, 100 and 200 μg/ml phillyrin protected cellular viability against the effect of ethanol by MTT assay, suggesting the potential efficacy of phillyrin (Supplementary Fig. 3B). Here, all the results led to the conclusion that phillyrin targets PDPK1 to inhibit the phosphorylation of PDPK1 (Ser241).

**Fig. 5:**
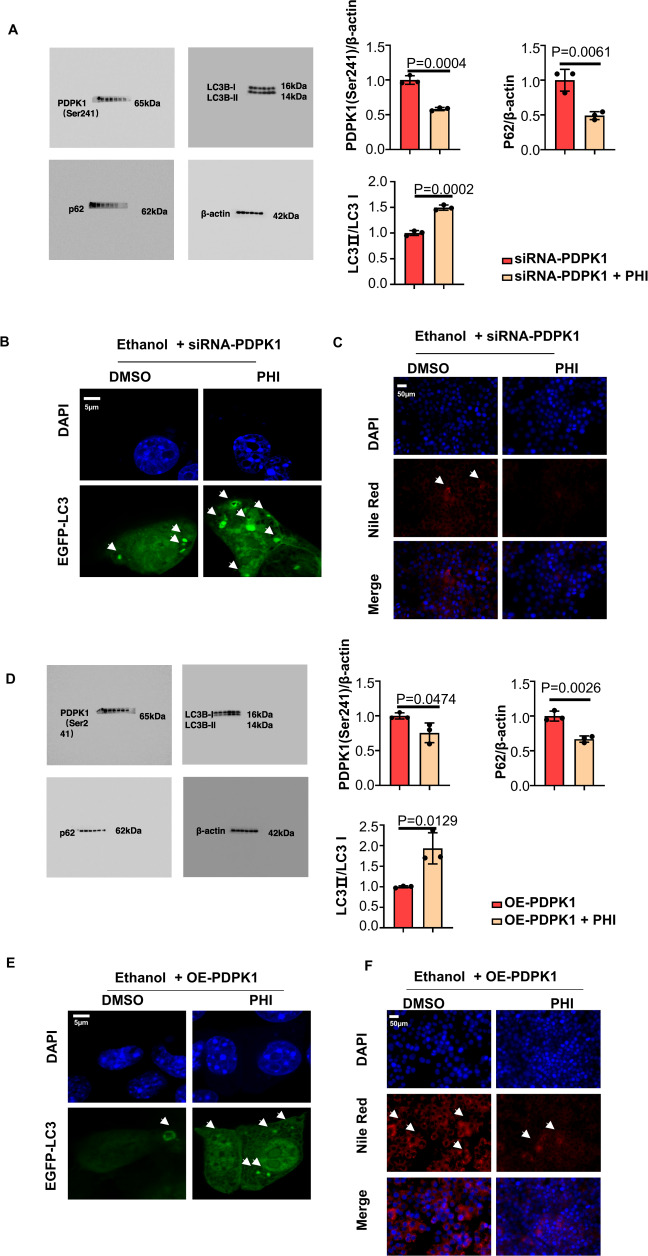
Phillyrin attenuated steatosis of hepatocytes by promoting autophagy by binding with PDPK1. **A** Immunoblot of cell lysates. Quantification of ratio of PDPK1 (Ser241)/β-actin, LC3II/LC3I and p62/β-actin (*n* = 3). **B** CLSM images of LC3 in AML-12 cells transfected with EGFP-LC3 plasmid (*n* = 3). Scale bar, 5 μM. **C** Nile red staining of lipid in AML-12 cells (*n* = 3). Scale bar, 50 μM, magnification, 400×. **D** Immunoblot of cell lysates. Quantification of the ratio of PDPK1 (Ser241)/β-actin, LC3II/LC3I and p62/β-actin (*n* = 3). **E** CLSM images of LC3 in AML-12 cells transfected with EGFP-LC3 plasmid (*n* = 3). Scale bar, 5 μM. **F** Nile red staining of lipid in AML-12 cells (*n* = 3). Scale bar, 50 μM, magnification, 400×. Data represent the mean ± SEM of three biological replicates per condition. Each dot represents a sample. *P* < 0 .05 was considered statistically significant. NS indicates no significance (**A** and **D**, two-tailed *t*-tests).

The effects of PDPK1 and phillyrin were investigated above. However, more experiments should be performed to test whether phillyrin attenuates ASH via PDPK1. When PDPK1 was downregulated by siRNA-PDPK1, the results of immunoblotting and CLSM demonstrated that phillyrin further decreased autophagy-flux in AML-12 cells (Fig. 5A, B). In addition, the decrease in Nile red-stained lipid droplets showed that phillyrin could further exert protective activities (Fig. 5C). Moreover, phillyrin inhibited the downregulation of the autophagic flux caused by HBLV-m-PDPK1-3xflag-mcherry-PURO in AML-12 cells (Fig. 5D, E). Fewer lipid vacuoles were observed in PDPK1 overexpression cells after treated with phillyrin (Fig. 5F).

### Phillyrin protected hepatocytes from suffering heavy alcoholic hepatitis by upregulating autophagy in vitro and in vivo

**Fig. 6:**
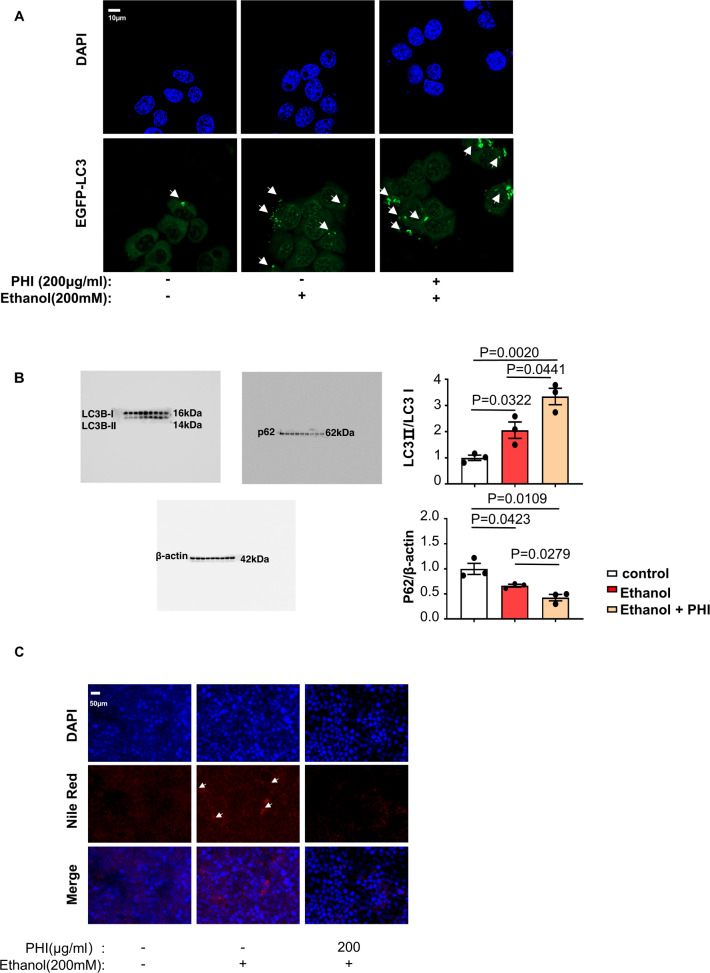
Philllyrin further promoted autophagic flux in ethanol-treated AML-12 cells. **A** CLSM images of LC3 in AML-12 cells transfected with EGFP-LC3 plasmid (*n* = 3). Scale bar, 10 μM. **B** Immunoblot of cell lysates from the control group, ethanol-induced group and ethanol-induced group treated with phillyrin (200 μg/ml) (3 representatives shown). Quantification of the ratios of LC3II/LC3I and p62/β-actin. **C** Nile red staining of lipids in AML-12 cells (*n* = 3). Scale bar, 50 μM, magnification, 400×. Data represent the mean ± SEM of three biological replicates per condition. Each dot represents a sample. *P* < 0.05 was considered statistically significant (B, one-way ANOVA).

Both the function of PDPK1 in autophagy and the interaction between phillyrin and PDPK1 were confirmed above, suggesting that phillyrin may alleviate the ethanol-induced injury via PDPK1-dependent autophagy. Next, a series of in vitro model experiments were performed to test our hypothesis. As expected, phillyrin induced autophagic flux in AML-12 cells. We found that ethanol-induced GFP-LC3 points were apparently enhanced by phillyrin (200 μg/ml) in AML-12 cells transfected with GFP-LC3 plasmid (Fig. 6A). Meanwhile, the increase in LC3II/LC3I and decrease in p62/β-actin in phillyrin-cultured AML-12 cells indicated that autophagy-flux had been induced, which was assayed by western blotting (Fig. 6B). Accordingly, Fig. 6C shows that intracellular lipid vacuoles were significantly decreased in the phillyrin (200 μg/ml) culture compared with the model. The efficacy of phillyrin was verified in an in vitro model, which attenuated the steatosis of AML-12 cells by promoting autophagy.

**Fig. 7:**
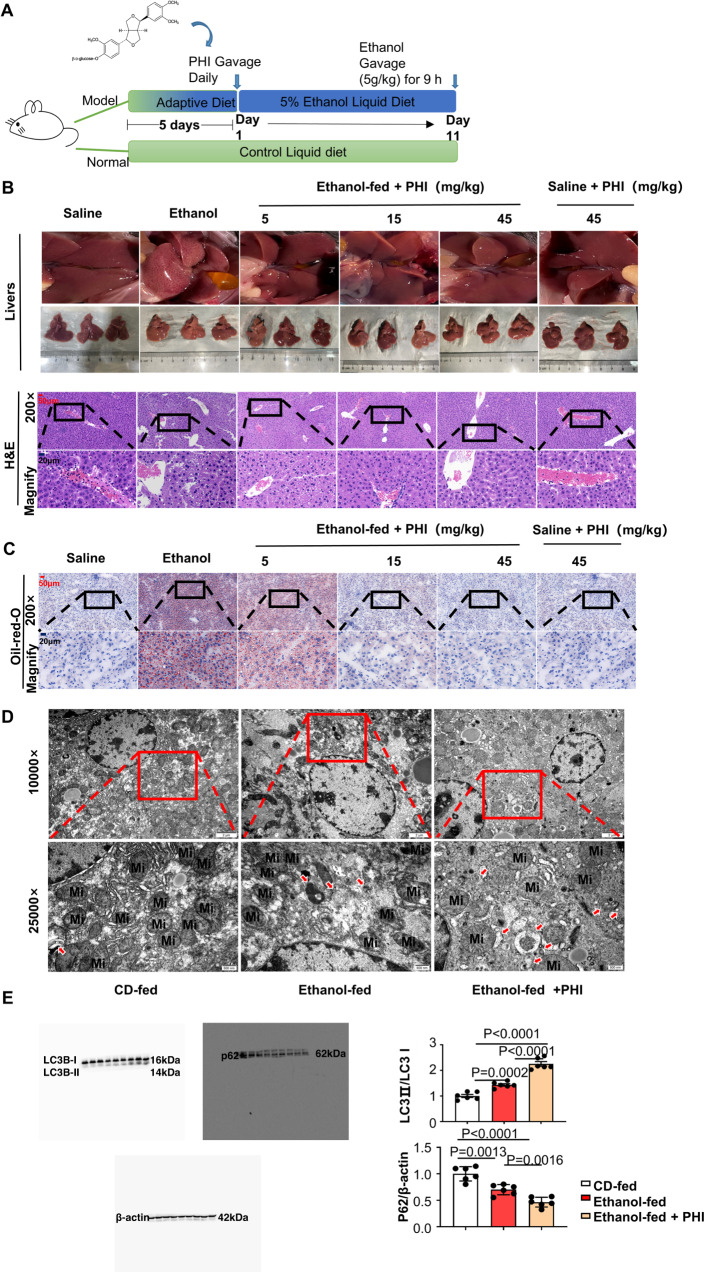
Phillyrin protected ethanol-fed mice from suffering heavy alcoholic hepatitis by upregulating autophagy. **A** The Gao-Binge protocol was used to establish an ASH model in C57BL/6 mice. Model mice were given a liquid diet adaptation for 5 days and a 5% ethanol-feeding diet for 10 days. Normal mice were given a control liquid diet for 15 days. (6–8 mice per group). Mice treated with the drug had been given phillyrin by daily gavage since Day 6. **B** H&E staining of liver tissues from different groups, including CD-fed mice, ethanol-fed mice, and 5, 15, and 45 phillyrin-treated model mice (*n* = 6). Scale bar, 50 μM, magnification, 200×; Magnified pictures, scale bar, 20 μM. **C** Oil red O staining of liver tissues from different groups, the pictures are sorted the same as the H&E staining (*n* = 6). Scale bar, 50 μM, magnification, 200×; Magnified pictures, scale bar, 20 μM. **D** Representative image of liver tissue captured by transmission electron microscopy (*n* = 6). Autophagosomes are marked by red arrows. Mi is the abbreviation of mitochondria. Scale bar, 2 μM, magnification, 1000×; scale bar, 500 nM, magnification, 2500×. **E** Immunoblot of liver homogenates of mice from the CD-fed group, ethanol-fed group and ethanol-fed group treated with phillyrin (45 mg/kg). Quantification of the ratios of LC3II/LC3I and p62/β-actin (*n* = 6). Data represent the mean ± SEM of six biological replicates per condition. Each dot represents a mouse. *P* < 0.05 was considered statistically significant (**E**, one-way ANOVA).

To further test phillyrin efficacy in vivo, we established an ASH animal model. Figure 7A shows the chemical structure of phillyrin and the method of establishing the ASH models by adopting the Gao-Binge protocol. The liver images and H&E staining of each group showed that injury was markedly reduced in liver tissues from phillyrin-treated mice with alcoholic hepatitis (Fig. 7B). Consistent with the above observations, oil red O staining showed that the number of lipid droplets was significantly decreased in phillyrin-treated mice (Fig. 7C). The levels of serum ALT and AST were significantly upregulated by ethanol, whereas phillyrin (15, 45 mg/kg) considerably attenuated the injury caused by ethanol (Supplementary Fig. 4A, B). Furthermore, the levels of serum TG and T-CHO were remarkably decreased in phillyrin-treated mice (Supplementary Fig. 4C, D). To determine whether phillyrin induces autophagic flux in vivo, TEM was performed using liver samples from mice. According to TEM images, the number of autophagic vacuoles (AVs), observed as double-membrane structures containing undigested cytoplasmic contents, was increased in the phillyrin-treated (45 mg/kg) mouse livers compared with the other two groups. Meanwhile, it seems that phillyrin increased the number of mitochondria and protected mitochondrial structure (Fig. 7D). Our researchers further proved this supposition by using CLSM and immunoblot (Supplementary Fig. 5A–C). Results of western blot also showed that phillyrin induced autophagy-flux (the level of LC3II/LC3I increased, p62/β-actin decreased) (Fig. 7E).

### Phillyrin targeted PDPK1 to suppress Akt/mTOR signalling

**Fig. 8:**
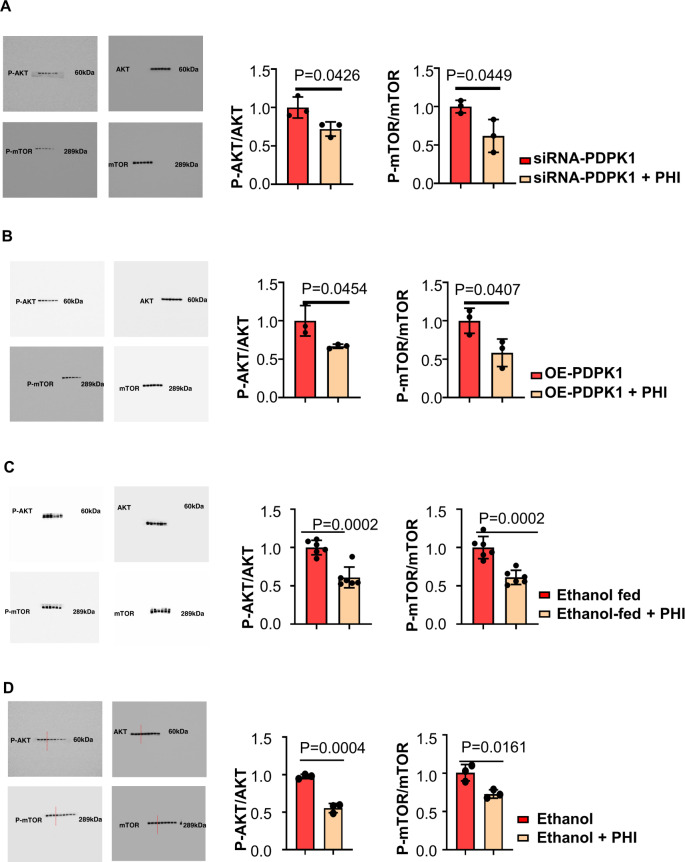
Phillyrin targets PDPK1 to suppress Akt/mTOR signalling. **A** Immunoblots of Akt/mTOR signalling in the two groups under ethanol stimulation: PDPK1 knockdown group treated with control and PDPK1 knockdown group treated with phillyrin (200 μg/ml). **B** Immunoblots of Akt/mTOR signalling in the two groups under ethanol stimulation: the PDPK1 overexpression group treated with DMSO as a control and the PDPK1 overexpression group treated with phillyrin (200 μg/ml). **C** Immunoblots of Akt/mTOR signalling in two groups: ethanol-fed mice and ethanol-fed mice treated with phillyrin (45 mg/kg). **D** Immunoblots of Akt/mTOR signalling in the two groups under ethanol stimulation: control group and group treated with phillyrin (200 μg/ml). Data represent the mean ± SEM of three or six biological replicates per condition. Each dot represents a sample. *P* < 0.05 was considered statistically significant (**A**–**D**, two-tailed *t*-tests).

The PDPK1/AKT/mTOR signalling cascade plays a key role in regulating autophagy [30]. We performed immunoblotting to analyse the levels of p-AKT (Ser473), AKT, p-mTOR (Ser2448) and mTOR under several experimental conditions. As PDPK1 has been proven to be the target of phillyrin, immunoblotting of p-AKT (Ser473), AKT, p-mTOR (Ser2448) and mTOR was performed to investigate the cascading effect of phillyrin targeting PDPK1. The ratios of p-AKT (Ser473)/AKT and p-mTOR (Ser2448)/mTOR were decreased by phillyrin not only compared with the PDPK1 knockdown group, but also the PDPK1 overexpression group (Fig. 8A, B). According to in vivo and in vitro experiments, the ratio of p-AKT (Ser473)/AKT and p-mTOR (Ser2448)/mTOR decreased in the phillyrin-treated group (Fig. 8C, D). Overall, these experiments revealed that phillyrin targets PDPK1 to suppress Akt/mTOR signalling.

## Discussion

Alcohol is widely consumed as a daily drink in many parts of the world due to the widespread popularity of drinking culture [31]. Unfortunately, multiple studies have indicated that alcohol can pose a serious threat to human health [4, 32]. Recently, the World Health Organisation (WHO) posted a series of striking numbers, and the death toll caused by harmful alcohol consumption is approximately 3,3 million (approximately 5,9% of global deaths) [33]. Alcoholic liver disease (ALD) is the leading cause of morbidity and mortality worldwide [34]. Alcoholic hepatic steatosis (ASH) is an important stage of the deteriorating course of ALD. However, the primary mechanism of the pathogenesis of ASH has not been clearly reported. Growing evidence indicates that the body triggers self-protection mechanisms when exposed to external stimuli [35, 36]. Therefore, we speculate that the effect of reducing body damage can be achieved by enhancing the body’s self-protection. Here, we demonstrated that PDPK1 played a significant role in regulating the cellular self-protection process of autophagy. Mechanistically, we not only discovered that PDPK1 was obviously expressed in ASH but also demonstrated that inhibiting PDPK1 and the phosphorylation of PDPK1 (ser241) could protect hepatocytes from suffering heavy alcoholic steatosis by upregulating autophagic flux for the first time. Pharmacologic inhibition of PDPK1 phosphorylation (Ser241) by targeting PDPK1 could provide an efficient treatment for future ASH healing.

Previous articles have reported that autophagy can be activated by acute alcohol stimulation in vivo and in vitro [37]. Autophagy is regarded as a highly conserved catabolic process, because it can wrap damaged organelles and proteins up and then bind with lysosomes to degrade and recycle cellular waste [38]. Since autophagy can alleviate or aggravate symptoms in different diseases or in different stages of one kind of disease, it is recognised as a double-edged sword [39]. The function of autophagy in ASH is still controversial. Therefore, we established an ASH animal model to investigate autophagy. Consistent with several reports, autophagy was upregulated in our ASH mice compared with normal mice. Our autophagy-inducer-treated mice suffered fewer ASH symptoms than saline-treated mice, although they were given the same diet. According to our results, autophagy could mitigate injury in ASH.

PDPK1 is a phosphorylation-regulated kinase that plays a central role in activating multiple signalling pathways and cellular processes [40]. It was reported that PDPK1 regulates autophagosome biogenesis [41]. In our ASH model, PDPK1 was observed to be markedly increased. We further tested the PDPK1-dependent autophagy mechanism by downregulating and upregulating PDPK1 in vitro. A series of experiments showed that PDPK1 blocked the process of cellular autophagy. In addition, the Ser241 phosphorylation of PDPK1 is necessary for phosphorylating AKT1 and activating the mTOR pathway [42], which negatively regulates autophagosome formation by phosphorylating ULK [43]. Fortunately, phillyrin was observed to bind with PDPK1 by SPR and CESTA experiments. Then, the analysis of the PHASE module of Schrödinger’s molecular modelling software package elucidated how phillyrin could target PDPK1 and how phillyrin influences the Ser241 phosphorylation of PDPK1. Later, the inhibition of the Ser241 phosphorylation of PDPK1 was proved in the Philllyrin-treated group. Thus, it is easily speculated that phillyrin may regulate autophagy by targeting PDPK1. Phillyrin is an extract from Forsythia suspensa that exerts several functions, including antibacterial, antiviral, anti-inflammatory, antioxidant, and other pharmacological effects [44–46]. We further explored the function of phillyrin by establishing an ASH model in vivo and in vitro.

In summary, we demonstrated a regulatory signalling pathway between PDPK1 and autophagy (the PDPK1/AKT/mTOR signalling pathway) and showed that both inhibiting PDPK1 and its Ser241 phosphorylation may contribute to ethanol resistance. Detecting the mechanism of PDPK1 in ethanol-induced hepatotoxicity will provide exciting new information for the treatment of ASH.

## Materials and methods

### Materials and reagents

Phillyrin was provided by Nanjing Biohybrid Pharmatech Co., Ltd. (CAS 487-41-2, Nanjing, China). The antibodies against mTOR, p-mTOR, AKT and p-AKT were purchased from Cell Signalling Technology (Danvers, MA, USA). Antibodies against LC3, p62 and TOMM20 were purchased from Abcam (Cambridge, UK). The recombinant human PDPK1 protein was also purchased from Abcam (Cambridge, UK). P-PDPK1(Ser241) antibody and goat anti-rabbit/mouse immunoglobulin G (IgG) horseradish peroxidase (HRP) secondary antibody were purchased from Abmart (Shanghai, China). β-actin antibody was purchased from Bioss (Beijing, China). An ALT/GPT (C009-2-1), AST/GOT (C010-2-1) assay kit, TG(A110-1-1) assay kit and T-CHO(A111-1-1) assay kit were purchased from Jiancheng Bioengineering Institution (Nanjing, China). Nile red was provided by Shanghai Yuanye Biotechnology Co., Ltd. (CAS 7385-67-3).

### Animal treatment

Male C57BL/6 mice that were 6–8 weeks old and that weighed >20 g were provided by the Laboratory Animal Centre of Anhui Medical University. All animal procedures were approved by the Institutional Animal Experimentation Ethics Committee of Anhui Medical University. These mice were maintained in the vivarium facility with 12 h light/dark cycles. The Gao-Binge protocol recommended by the National Institute on Alcohol Abuse and Alcoholism (NIAAA) was adopted to establish the ASH model. In this protocol, mice were given a liquid diet adaptation for 5 days and an ethanol-feeding diet for 10 days. On the last day, mice were treated with a single binge ethanol administration (5 g/kg, body weight, 20% ethanol) by gavage. CD-fed mice were fed control liquid diets and treated with isocaloric maltose-dextrin by gavage on the last day. All diets were prepared fresh daily. Nine hours after the last alcohol gavage, the mice were euthanized, and the liver tissues and blood were collected for further analysis.

### Liver histological analysis

Then, 4% paraformaldehyde fixative was used to immerse part of the hepatic lobes cut from C57BL/6 J mice for 48 h. Then, paraffin was used to embed the samples. Next, 5-μm thick sections were subjected to haematoxylin and eosin (H&E) and immunohistochemical (IHC) staining of PDPK1. Livers embedded in optimum cutting temperature compound were used for oil red O staining for the assessment of hepatic steatosis. The sections were finally scanned under SlideViewer (3DHISTECH Ltd.).

### Serum levels of ALT/GPT, AST/GOT1, TG and T-CHO analysis

Serum ALT/GPT, AST/GOT, TG and T-CHO levels were assayed using GPT, GOT1, TG and T-CHO activity assay kits (Nanjing Jiancheng Bioengineering). The absorbance was measured at 510 nm (GPT, GOT, TG, T-CHO) with a Cytation5 Cell Imaging Microplate Assay System (Bioteck, US).

### Electron microscopy

Mouse livers were fixed with a combination of paraformaldehyde and glutaraldehyde, and then livers were processed and sectioned with a diamond knife on copper grids after fixation. Grids were examined with a Hitachi (Tokyo, Japan) 7100 electron microscope, and images were captured using a MegaView III digital camera (Soft Imaging System, USA).

### Cell culture

AML-12 cells were obtained from the Chinese Academy of Sciences (Shanghai, China), cultured in DMEM F12 (Procell, China) supplemented with 10% FBS (Gibco, US) and incubated at 37 °C in an atmosphere of 5% CO_2_. Then, the cells were cultured by adding 200 mM ethanol for 24 h or incubating with different concentrations of phillyrin(50, 100, 200 μg/ml) while adding 200 mM ethanol for 24 h.

### MTT assay

We measured the safe dose of philyrin by MTT assay. AML-12 cells were seeded in 96-well plates, and the edge wells were filled with sterile PBS. After adherence, cells were cultured with various concentrations of phillyrin for 24 h. A total of 20 μL of 5 mg/mL MTT was added to each well and incubated with the cells at 37 °C for 4–6 h. After the removal of the supernatant, 150 μL of DMSO was added to each well. The optical density (OD) was measured at 490 nm. The percent of viable cells was calculated according to the formula.

### EGFP-LC3 transient transfection

AML-12 cells were cultured on the surface of the coverslip stably first. Then those cells were transfected with the EGFP-LC3 plasmid for 24 h using the transfection mate (GenePharma, China). After transfection for 6 h, the cells were treated with phillyrin for 24 h. Subsequently, the cells were stained with DAPI (Bioss, China) and observed with a Zeiss LSM880 confocal laser microscopy system and analysed using the Zeiss LSM Browser.

### Western blotting

Proteins from liver tissue (30 or 50 mg) and AML-12 cells were extracted with RIPA lysis buffer (Beyotime, China) with 1% phenyl methyl sulfonyl fluoride (PMSF; Beyotime), and the protein concentration was measured with a BCA protein assay kit (Beyotime, China) according to the manufacturer’s instructions. Proteins were separated by sodium dodecyl sulfate‒polyacrylamide gel electrophoresis (SDS‒PAGE) and transferred to PVDF membranes (size of 0.22 μm just for LC3, size of 0.45 μm for other proteins, Millipore Corp, Billerica, MA, USA). The PVDF membranes were blocked in 5% skim milk for 1.5 h at room temperature and then, washed three times in TBST. Subsequently, the PVDF membranes were incubated with primary antibodies against PDPK1 (1:1000), β-actin (1:1000), LC3 (1:1000), p62 (1:1000), p-AKT (1:2000), AKT (1:1000), P-MTOR (1:1000), and mTOR (1:1000) overnight at 4 °C, followed by incubation with secondary antibodies (1:5000) for 1 h at room temperature. The protein bands were visualised with an ECL-chemiluminescent kit (Epizyme, China). All experiments were repeated three times.

### RNA interference analysis

Small interfering RNA (siRNA) oligonucleotides against the PDPK1 gene and scrambled sequences were designed and synthesised by HanzBio (Shanghai, China). The siRNA sequences were as follows:

PDPK1-siRNA (sense, 5ʹ-GCAACAUAGAGCAGUACAUTT-3ʹ and antisense, 5ʹ-AUGUACUGCUCUAUGUUGCTT-3ʹ);

Scrambled-siRNA (sense, 5ʹ-UUCUCCGAACGUGUCACGUTT-3ʹ and antisense, 5ʹ-ACGUGACACGUUCGGAGAATT-3ʹ).

AML-12 cells were transfected with 1000 ng/mL PDPK1-siRNA or scrambled-siRNA and mixed with Lipo3000 transfection reagent (Hanbio, China) according to the manufacturer’s instructions. After 6 h, the Opti-MEM was replaced by DMEM F12 supplemented with 10% FBS DMEM, and the cells were treated with 200 mM ethanol. The silencing efficiency was determined by RT‒qPCR.

### RNA extraction and quantitative real-time PCR analysis

Total RNA was extracted by using TRIzol total RNA isolation reagent (Invitrogen, Carlsbad, CA, USA). One microgram of total mRNA was used for reverse transcription with the Takara RT‒qPCR synthesis kit (Takara, Dalian, China), according to the manufacturer’s instructions. cDNA synthesis was performed using SYBR Premix Ex Taq II (Takara) on a PikoReal 96 qPCR system (Thermo Fisher Scientific, Waltham, MA, USA). The primer sequences used were as follows:

GAPDH forwards, 5′-AGGTCGGTGTGAACGGATTTG-3´;

GAPDH reverse, 5′-GGGGTCGTTGATGGCAACA-3´;

PDPK1 forwards, 5′-CTGTATGACGCTGTGCCCATT-3´;

PDPK1 reverse, 5′-AAGGGGTTGGTGCTTGGTC-3´.

GAPDH was used to normalise the expression values of the other genes. All experiments were repeated at least three times.

### Construction of a stable PDPK1-overexpressing AML-12 cell line

HBLV-m-PDPK1-3xflag-mCherry-PURO was provided by HanzBio (Shanghai, China). The multiplicity of infection (MOI) was detected first (MOI = 30). To generate stable PDPK1 overexpression cell populations, AML-12 cells were infected with HBLV-m-PDPK1-3xflag-mcherry-PURO or HBLV-mCherry-PURO-Control for 24 h. Then, fresh DMEM F12 with puromycin (0.5 μmg/ml) was added to plates to stably transfect cells for 48 h.

PDPK1 overexpression in the stable cell line was verified by RT‒qPCR.

### Surface plasmon resonance (SPR) technology-based assay

The surface plasmon resonance (SPR) method was used with a BIAcore T200 (Cytiva) to measure the binding affinities. During the experiment, PDPK1 was immobilised on a CM5 sensor chip according to the Biacore methods. Phillyrin (20 mM) was diluted with 5% DMSO Running Buffer [1×PBS-P+] to different concentrations as follows: 1000, 333.3, 111.1, 37, 12.3, 4.1, 1.37, 0.46, 0.15 and 0.05 μM. A random concentration was double used. Samples were injected into the channels at a flow rate of 30 μL/min and then, washed with HBS buffer. The binding RU (Response Unit) values of phillyrin to PDPK1 were recorded directly by the Biacore T200 instrument and analysed by Biacore T200 Evaluation Software.

### Cellular thermal shift assay (CETSA)

AML-12 cells were incubated with or without phillyrin (200 μg/ml) for 24 h. Then, the incubated cells were collected and resuspended to 1 × 10^7^ cells/ml with PBS. The cell suspension was divided into 10 parts, which were heated for 3 min at different temperatures (43, 46, 49, 52, 55, 58, 61, 64, 67 and 70 °C). The heated cells were kept at −80 °C for 12 h and then at room temperature for 5 min. This freezing and thawing process was repeated three times. After that, cell lysates were extracted by centrifugation at 20,000×*g* for 20 min. The levels of PDPK1 were assessed by western blotting.

### Statistical analysis

Differences in multiple groups were determined by one-way ANOVA analysis. Tukey’s post hoc test was performed after a one-way ANOVA analysis. However, differences between the two groups were determined by Student’s *t*-tests (unpaired, two-tailed). Experimental data in this study were analysed by using Prism 8.0 GraphPad Software (USA). *P* < 0 .05 was considered statistically significant.

## Supplementary information

A reproducibility checklist

Supplementary figure legends

Supplementary figure 1

Supplementary figure 2

Supplementary figure 3

Supplementary figure 4

Supplementary figure 5

Original Data File

## Data Availability

Other data that support the findings of this study are available from the corresponding author upon reasonable request.
