# Supplementary material for: Downregulating PDPK1 and taking phillyrin as PDPK1-targeting drug protect hepatocytes from alcoholic steatohepatitis by promoting autophagy
Source: Cell Death Dis. 2022 Nov 23;13(11):991. doi: 10.1038/s41419-022-05422-3 (PMC9684571; doi:10.1038/s41419-022-05422-3)
Supplement: Supplementary file 2 — Supplementary figure legends [file 41419_2022_5422_MOESM2_ESM.docx]

**Supplementary Figure Legends**

**Figure S1:** Serum indices (serum AST, ALT, TG, T-CHO) in different groups (CD-fed group, ethanol-fed group, ethanol-fed group treated with rapamycin (1 mg/kg).

(A) Serum AST in CD-fed group and ethanol-fed group (n=6).

(B) Serum ALT in CD-fed group and ethanol-fed group (n=6).

(C) Serum TG in CD-fed group and ethanol-fed group (n=6).

(D) Serum T-CHO in CD-fed group and ethanol-fed group (n=6).

(E) Serum AST in saline-treated mice and rapamycin-treated mice (n=6).

(F) Serum ALT in saline-treated mice and rapamycin-treated mice (n=6).

(G) Serum TG in saline-treated mice and rapamycin-treated mice (n=6).

(H) Serum T-CHO in saline-treated mice and rapamycin-treated mice (n=6).

Data represent the mean ± SEM of 6 biological replicates per condition. Each dot represents a mouse. P <0.05 was considered statistically significant. NS indicates no significance (two-tailed t tests).

**Figure S2:** The most suitable concentration of Ethanol for in vitro model.

Cell viability detected by MTT assay after AML-12 cells seeded in 96-well plate and treated with ethanol (0, 6.25, 12.5, 25, 50, 100, 200, 400, 800, 1600 mM) for 24h. (n=3).

Data represent the mean ± SEM of 3 biological replicates per condition. Each dot represents a sample. P <0.05 was considered statistically significant. NS indicates no significance (one-way ANOVA).

**Figure S3：**Cytotoxicity and efficacy of phillyrin.

(A) The cell viability detected by MTT assay after AML-12 cells had seeded in 96-well plate and treated with different gradients of phillyrin(0, 1.5625, 3.125, 6.25, 12.5, 25, 50, 100, 200, >200 μg/ml, phillyrin precipitated at higher concentration, n=3).

(B) The effectiveness of phillyrin (50, 100 200 μg/ml) was tested under 200 mM ethanol (n=3).

Data represent the mean ± SEM of 3 biological replicates per condition. Each dot represents a sample. P <0.05 was considered statistically significant. NS indicates no significance (one-way ANOVA).

**Figure S4：**Serum indices (serum AST, ALT, TG, T-CHO) in different groups.

Serum AST (A), ALT (B), TG (C), T-CHO (D) from different groups, including CD-fed mice, ethanol-fed mice, and 5 mg/kg, 15 mg/kg, and 45 mg/kg phillyrin-treated model mice (n=6).

Data represent the mean ± SEM of 6 biological replicates per condition. Each dot represents a mouse. P <0.05 was considered statistically significant. NS indicates no significance (one-way ANOVA).

**Figure S5：**Phillyrin protects mitochondria against the injury of ethanol.

1. CLSM images of mitochondria in AML-12 cells. Scale bar, 5 μM. Quantification of the ratio of mitochondria area/cell nuclear area (n=3).
2. Immunoblot of cell lysates. Quantification of the ratio of TOMM20/β-actin (n=3).
3. Immunoblot of liver tissue. Quantification of the ratio of TOMM20/β-actin (n=6).

Data represent the mean ± SEM of 3 or 6 biological replicates per condition. Each dot represents a sample. P <0.05 was considered statistically significant (one-way ANOVA).
