# Supplementary figures and images for: Downregulating PDPK1 and taking phillyrin as PDPK1-targeting drug protect hepatocytes from alcoholic steatohepatitis by promoting autophagy
Source: Cell Death Dis. 2022 Nov 23;13(11):991. doi: 10.1038/s41419-022-05422-3 (PMC9684571; doi:10.1038/s41419-022-05422-3)

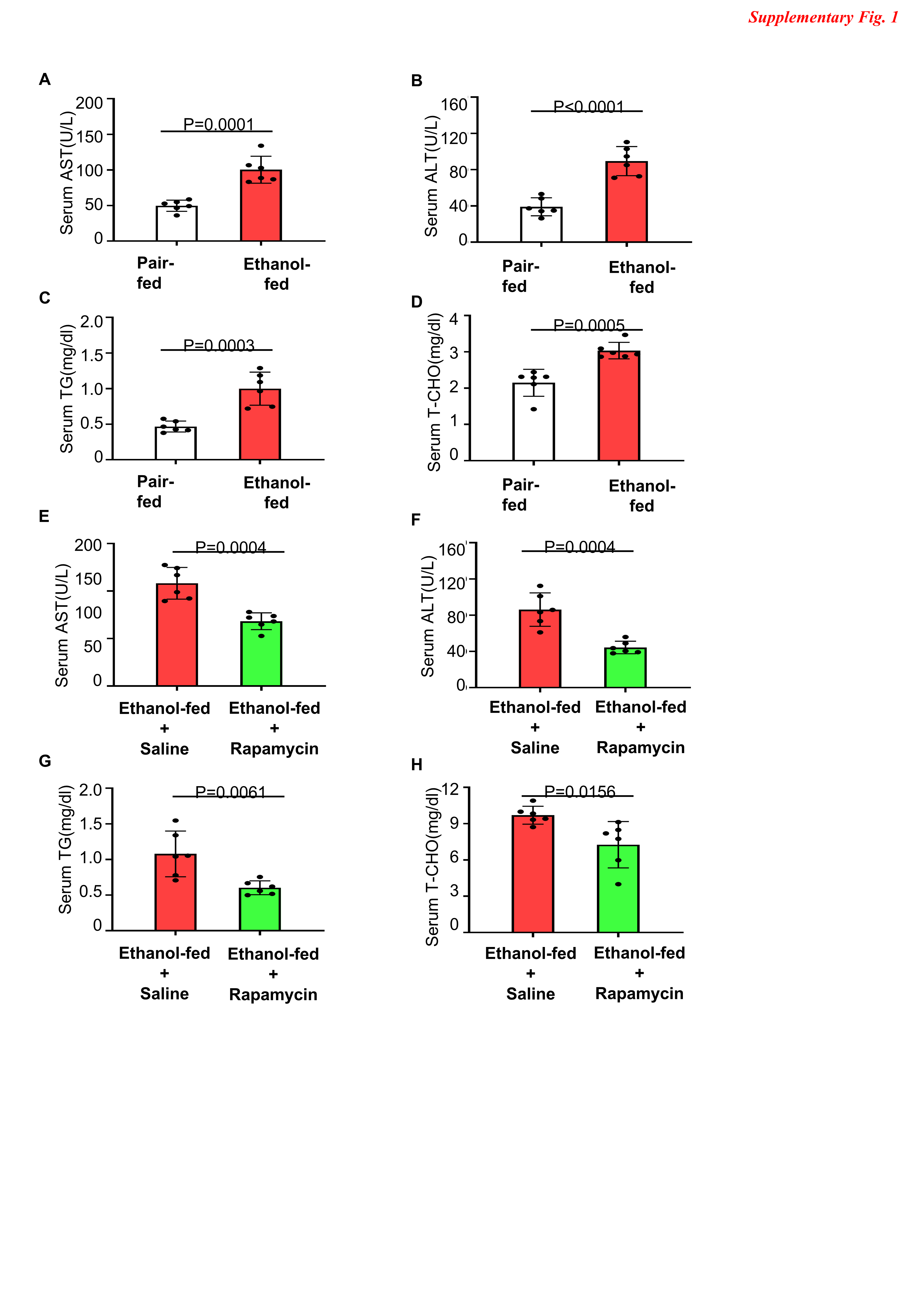

Supplement: Supplementary file 3 — Supplementary figure 1 [file 41419_2022_5422_MOESM3_ESM.tif]

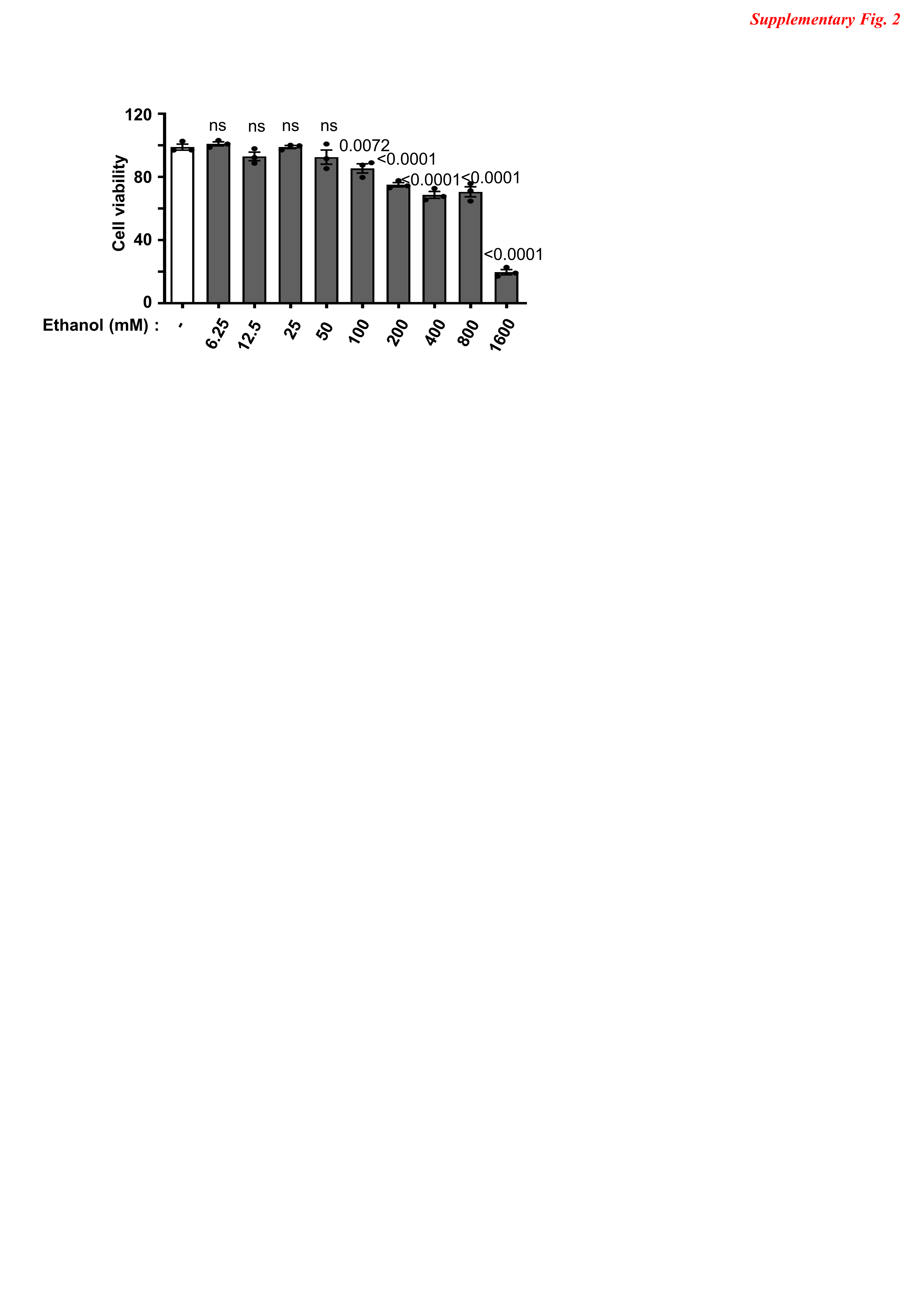

Supplement: Supplementary file 4 — Supplementary figure 2 [file 41419_2022_5422_MOESM4_ESM.tif]

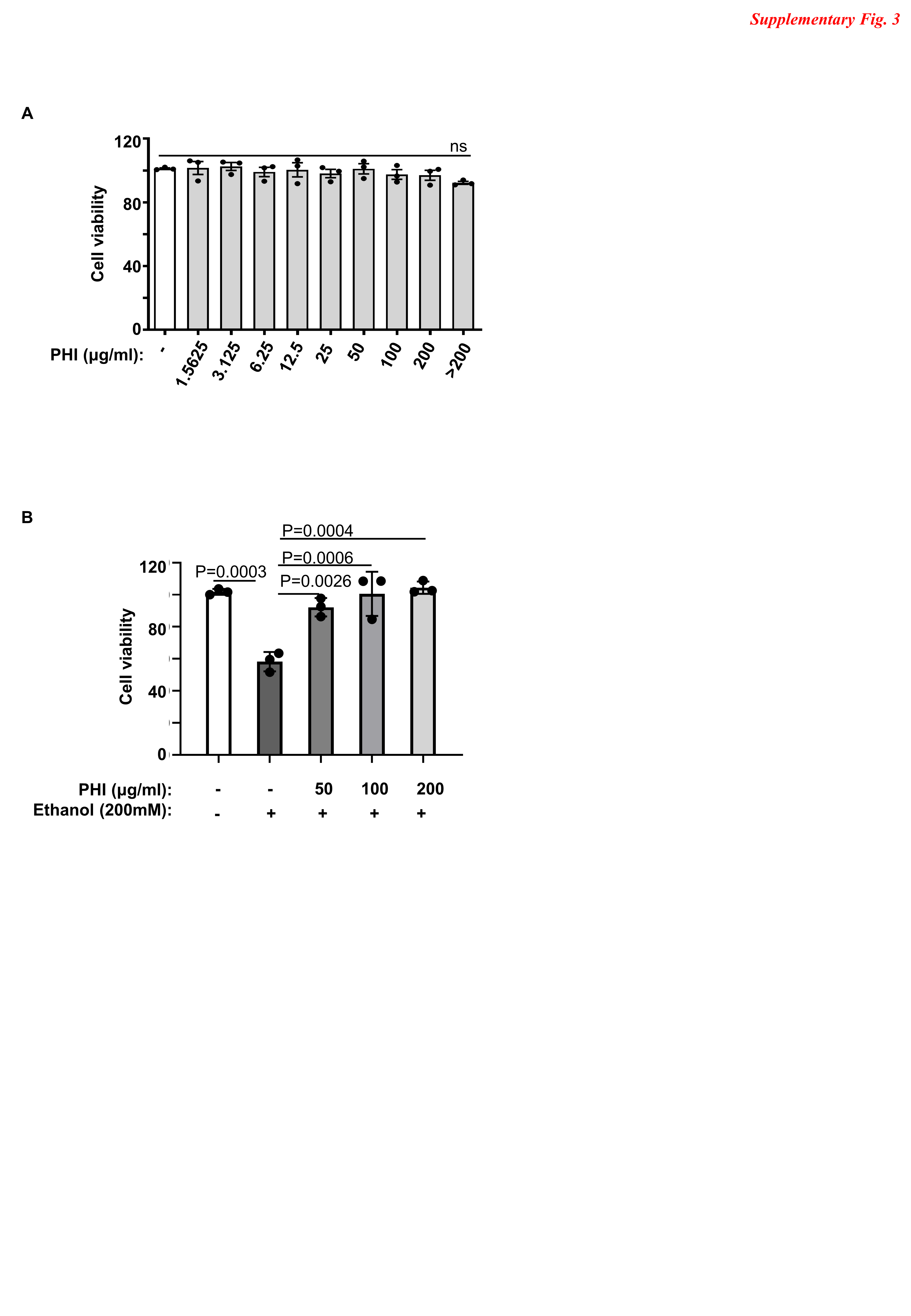

Supplement: Supplementary file 5 — Supplementary figure 3 [file 41419_2022_5422_MOESM5_ESM.tif]

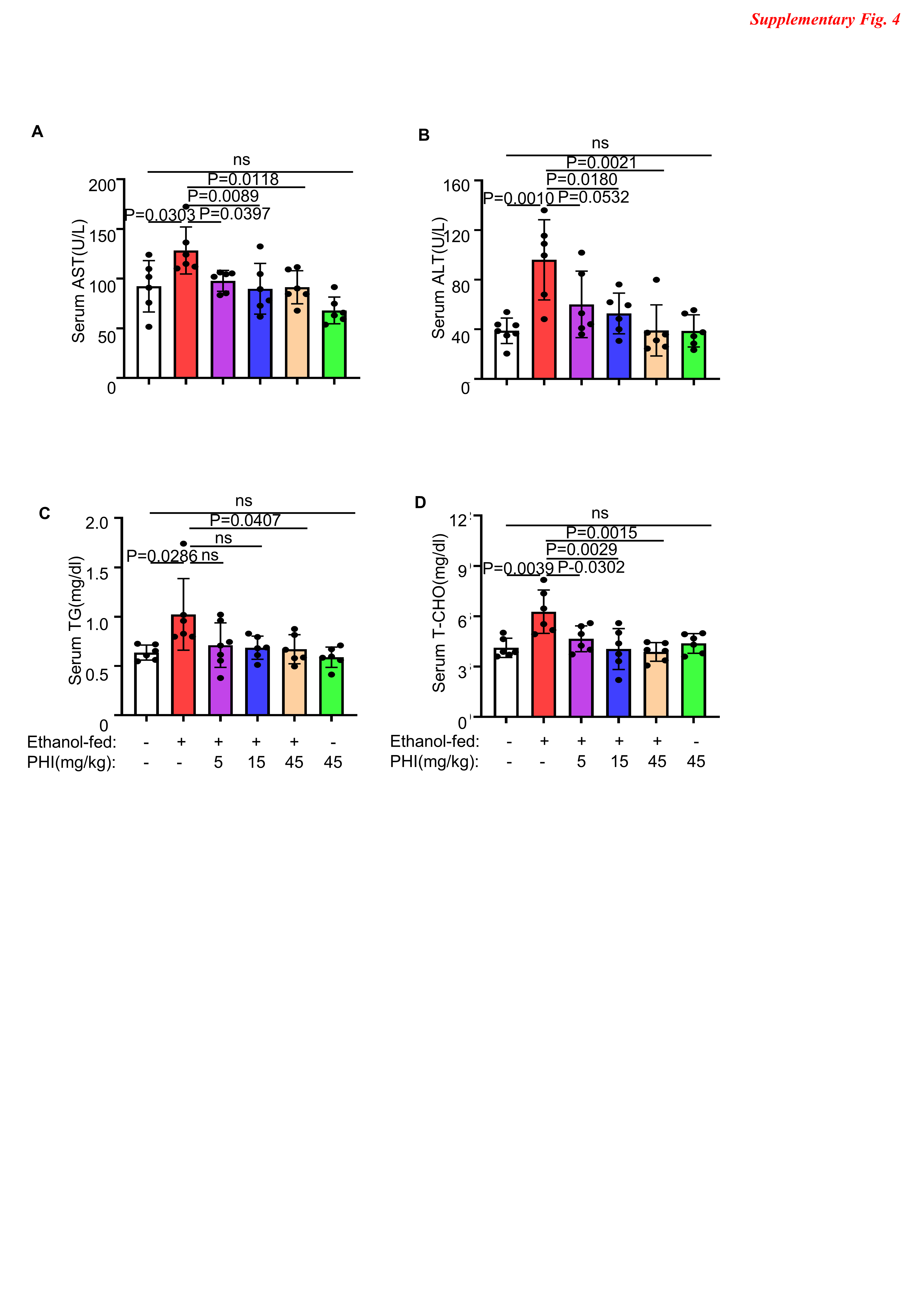

Supplement: Supplementary file 6 — Supplementary figure 4 [file 41419_2022_5422_MOESM6_ESM.tif]

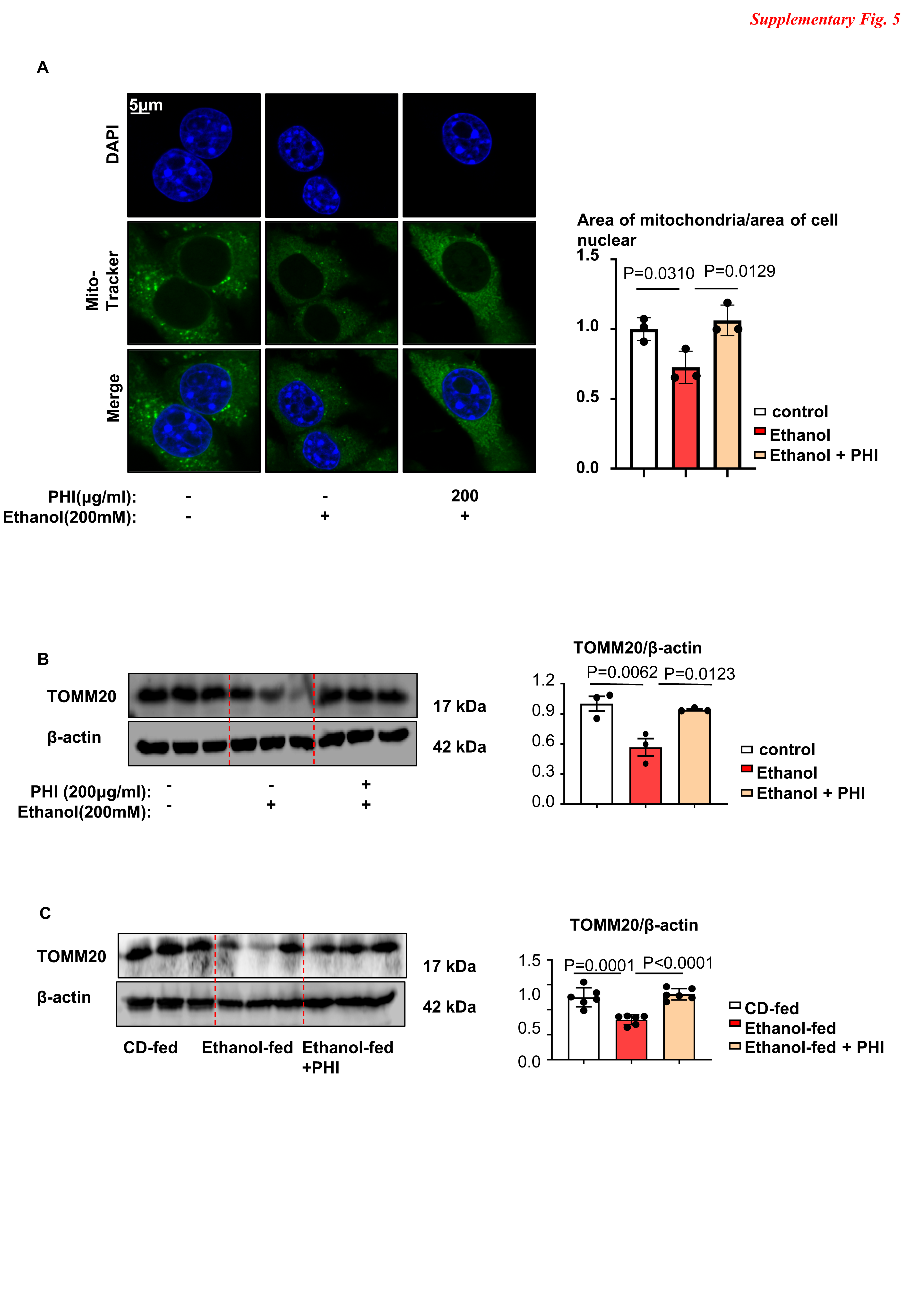

Supplement: Supplementary file 7 — Supplementary figure 5 [file 41419_2022_5422_MOESM7_ESM.tif]
