# Supplementary material for: Downregulating PDPK1 and taking phillyrin as PDPK1-targeting drug protect hepatocytes from alcoholic steatohepatitis by promoting autophagy
Source: Cell Death Dis. 2022 Nov 23;13(11):991. doi: 10.1038/s41419-022-05422-3 (PMC9684571; doi:10.1038/s41419-022-05422-3)

FIG. 1D

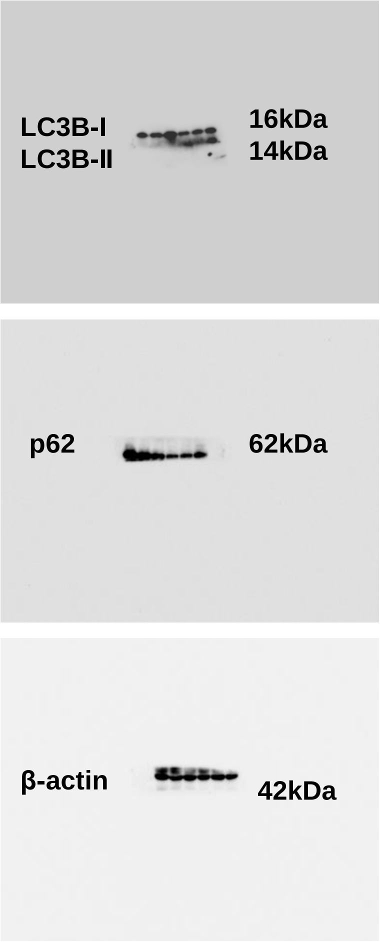
LC3B-I
LC3B-II
16kDa
14kDa
p62
62kDa
β-actin
42kDa

FIG. 1I

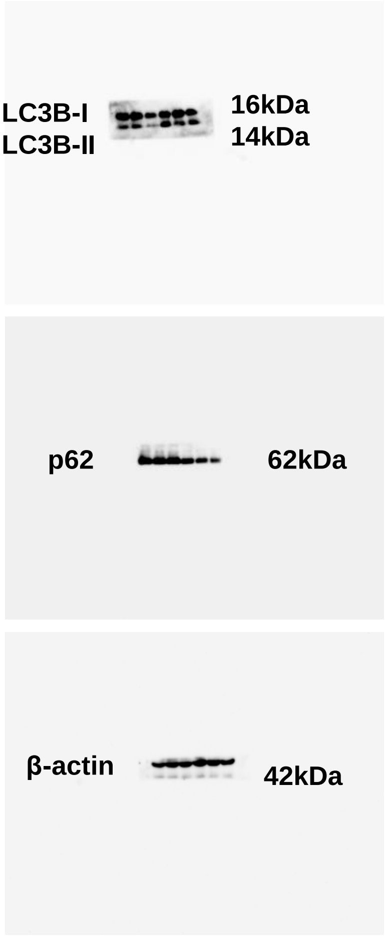
LC3B-I
LC3B-II
16kDa
14kDa
p62
62kDa
β-actin
42kDa

FIG. 2B

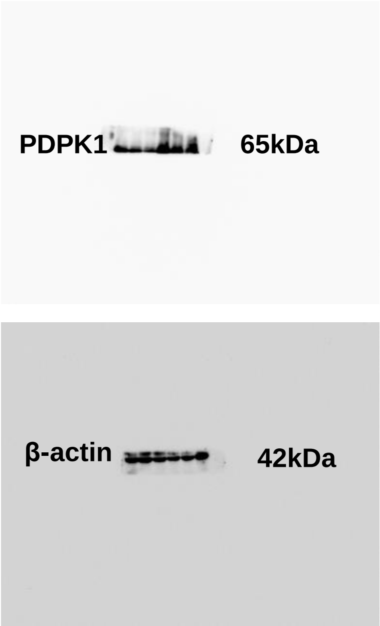

FIG. 2D

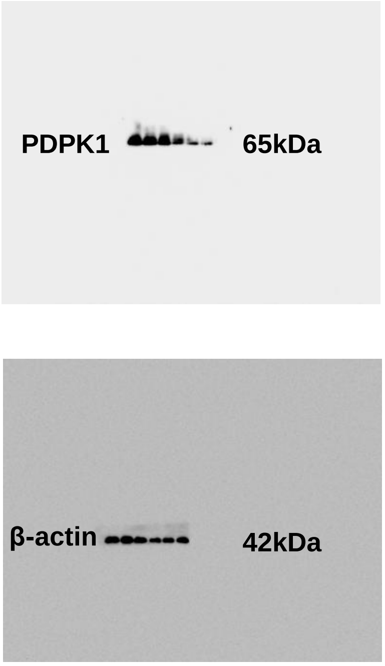

FIG. 2E

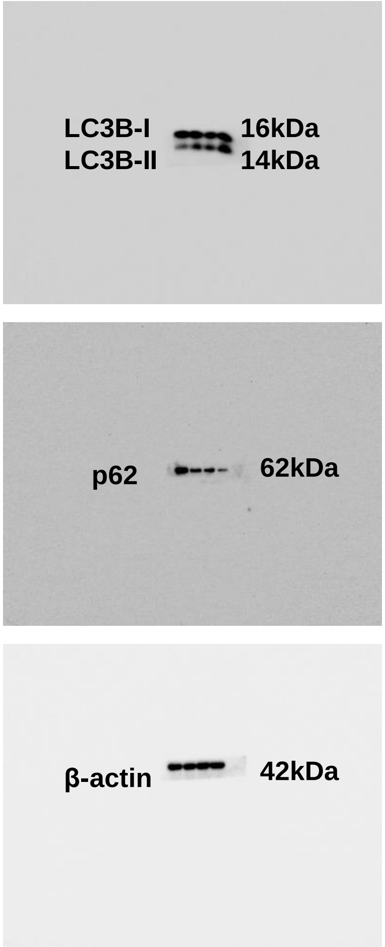

FIG. 3B

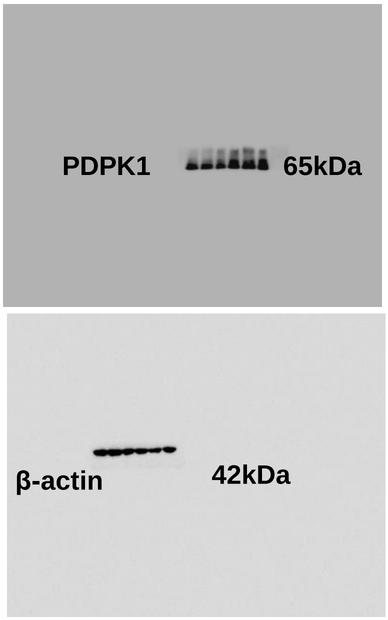
PDPK1
65kDa
β-actin
42kDa

FIG. 3C

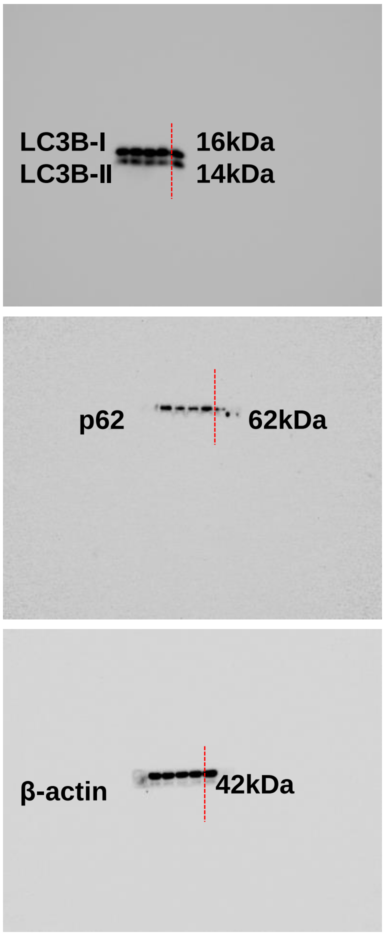
LC3B-I
LC3B-II
16kDa
14kDa
p62
62kDa
β-actin
42kDa

FIG. 4B

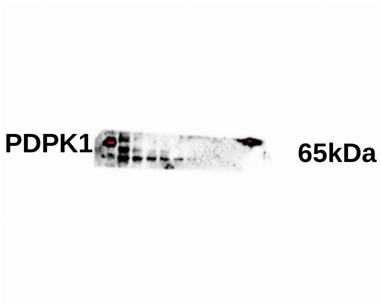

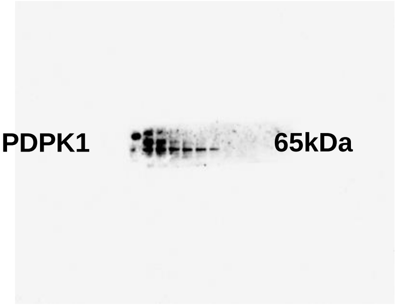

FIG. 4E

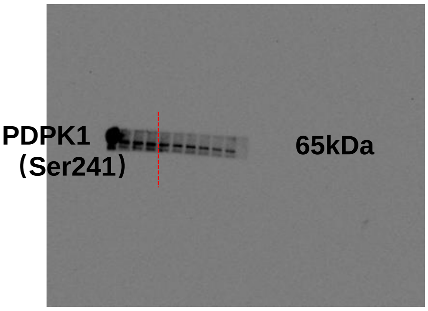

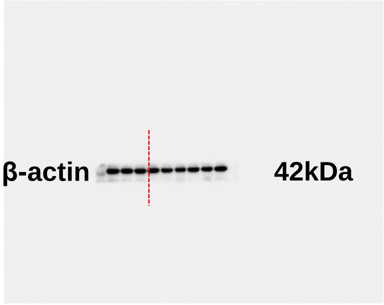

FIG. 5A

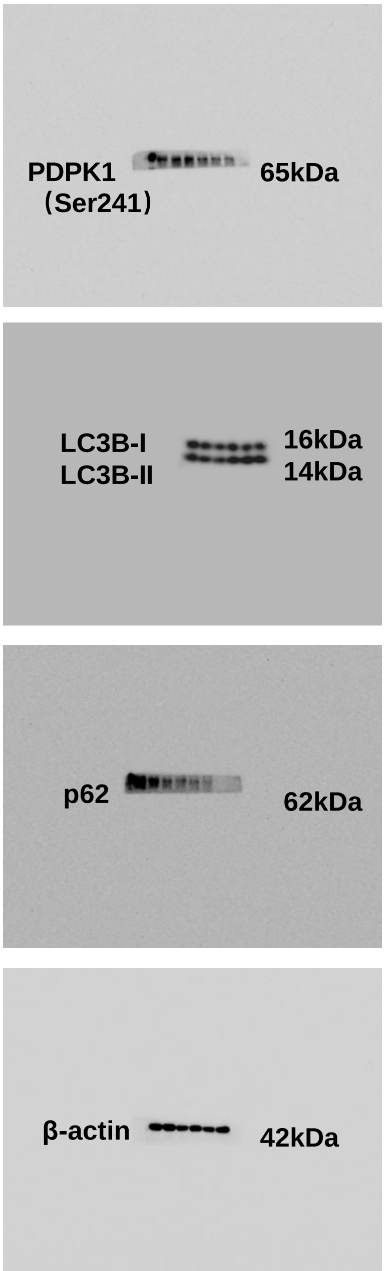
PDPK1
(Ser241)
65kDa
LC3B-I
LC3B-II
16kDa
14kDa
p62
62kDa
β-actin
42kDa

FIG. 5D

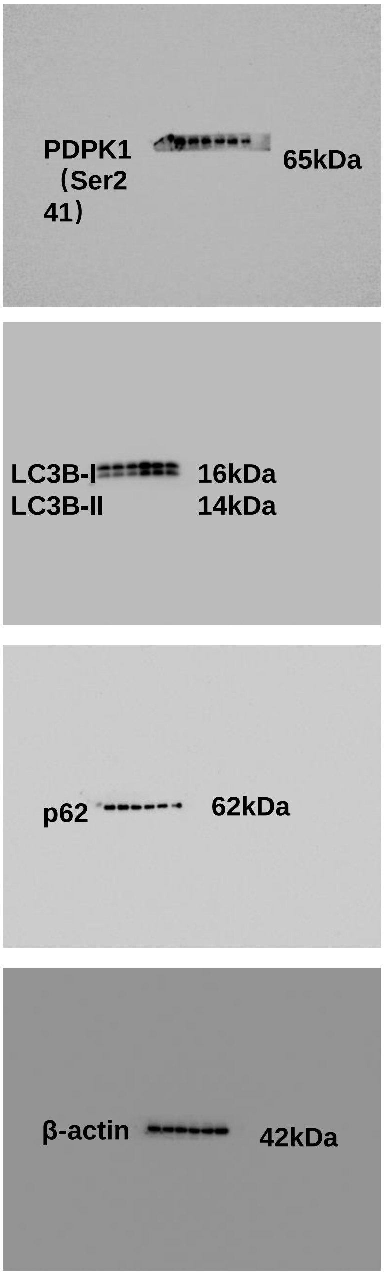
PDPK1
(Ser2
41)
65kDa
LC3B-I
LC3B-II
16kDa
14kDa
p62
62kDa
β-actin
42kDa

FIG. 6B

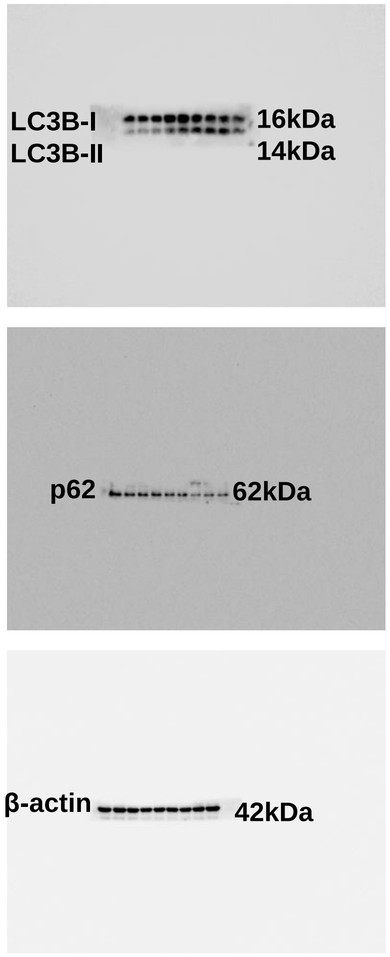

FIG. 7E

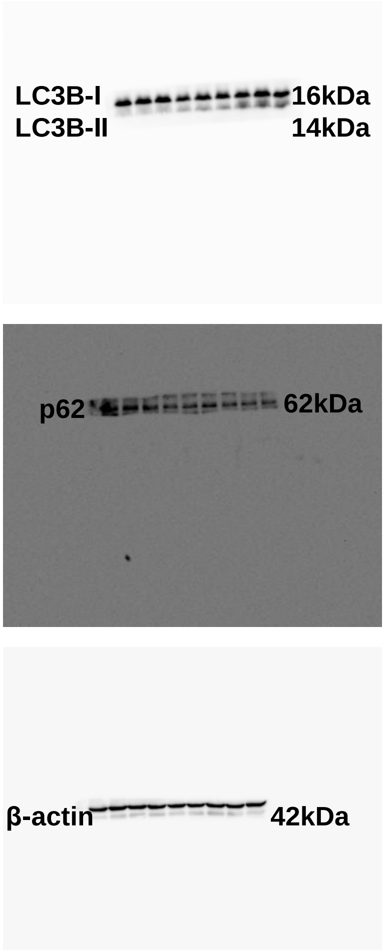

FIG. 8A

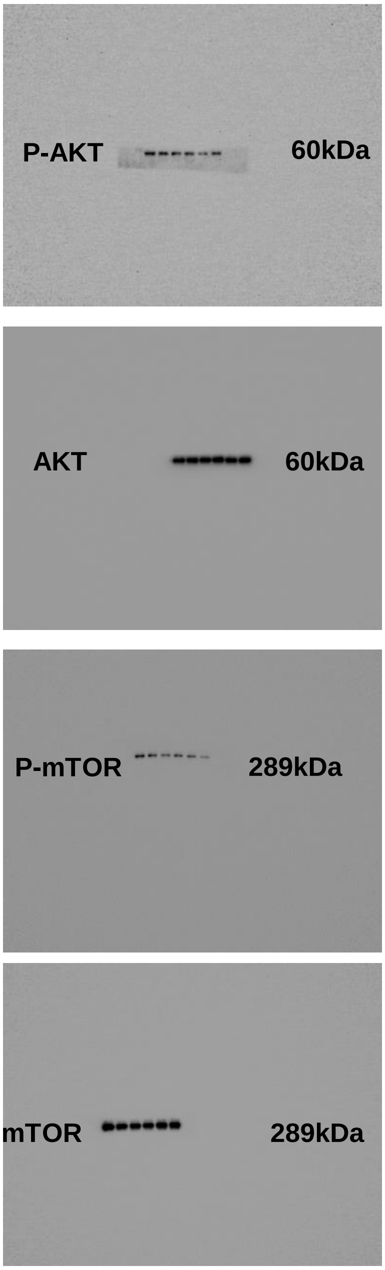

P-AKT
60kDa
AKT
60kDa
P-mTOR
289kDa
mTOR
289kDa

FIG. 8B

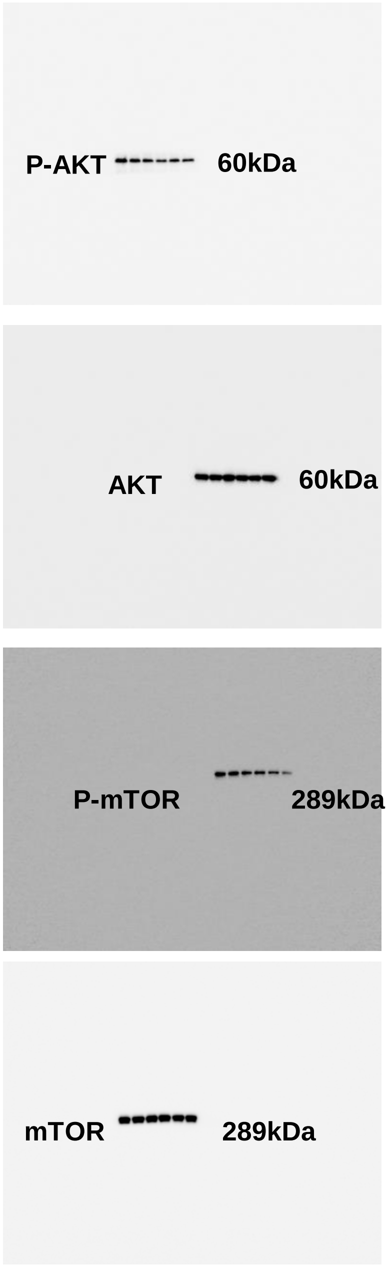

P-AKT
60kDa
AKT
60kDa
P-mTOR
289kDa
mTOR
289kDa

FIG. 8C

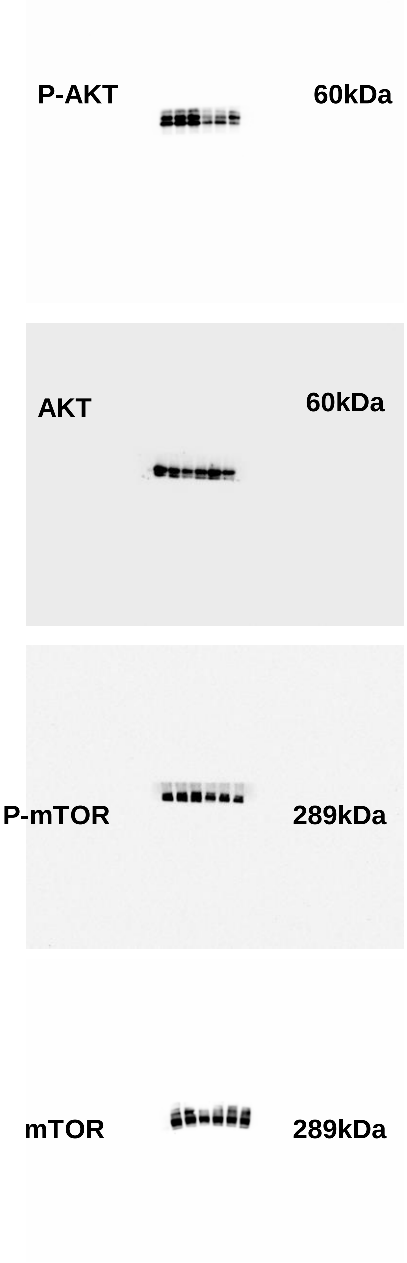
P-AKT
60kDa
AKT
60kDa
P-mTOR
289kDa
mTOR
289kDa

FIG. 8D

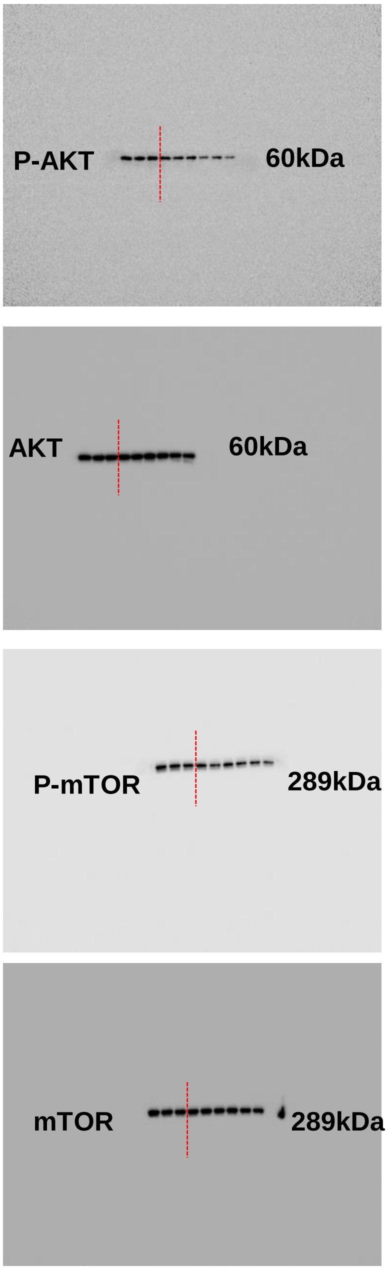
P-AKT
60kDa
AKT
60kDa
P-mTOR
289kDa
mTOR
289kDa

Supplementary figure 5B

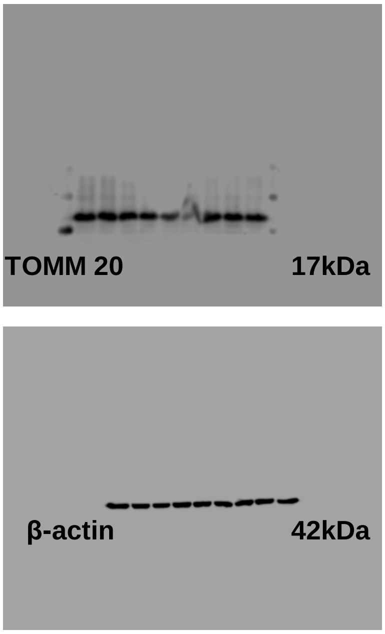

Supplementary figure 5C

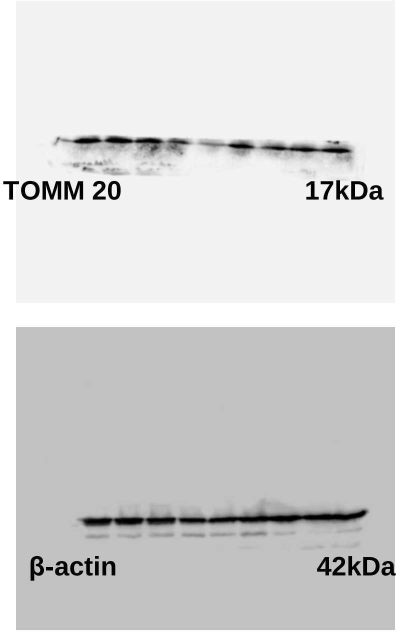

Supplement: Supplementary file 8 — Original Data File [file 41419_2022_5422_MOESM8_ESM.pdf]
